# Energy efficient photonic memory based on electrically programmable embedded III-V/Si memristors: switches and filters

**DOI:** 10.1038/s44172-024-00197-1

**Published:** 2024-03-18

**Authors:** Stanley Cheung, Bassem Tossoun, Yuan Yuan, Yiwei Peng, Yingtao Hu, Wayne V. Sorin, Geza Kurczveil, Di Liang, Raymond G. Beausoleil

**Affiliations:** 1 Hewlett Packard Enterprise, Large-Scale Integrated Photonics Lab, Milpitas, CA 95035 USA; 2 University of Michigan, Department of Electrical and Computer Engineering, Ann Arbor, MI 48109 USA

**Keywords:** Silicon photonics, Electrical and electronic engineering

## Abstract

Over the past few years, extensive work on optical neural networks has been investigated in hopes of achieving orders of magnitude improvement in energy efficiency and compute density via all-optical matrix-vector multiplication. However, these solutions are limited by a lack of high-speed power power-efficient phase tuners, on-chip non-volatile memory, and a proper material platform that can heterogeneously integrate all the necessary components needed onto a single chip. We address these issues by demonstrating embedded multi-layer HfO_2_/Al_2_O_3_ memristors with III-V/Si photonics which facilitate non-volatile optical functionality for a variety of devices such as Mach-Zehnder Interferometers, and (de-)interleaver filters. The Mach-Zehnder optical memristor exhibits non-volatile optical phase shifts > π with ~33 dB signal extinction while consuming 0 electrical power consumption. We demonstrate 6 non-volatile states each capable of 4 Gbps modulation. (De-) interleaver filters were demonstrated to exhibit memristive non-volatile passband transformation with full set/reset states. Time duration tests were performed on all devices and indicated non-volatility up to 24 hours and beyond. We demonstrate non-volatile III-V/Si optical memristors with large electric-field driven phase shifts and reconfigurable filters with true 0 static power consumption. As a result, co-integrated photonic memristors offer a pathway for in-memory optical computing and large-scale non-volatile photonic circuits.

## Abstract (web summary)

Stanley Cheung and co-authors introduce co-integrated III-V/Si memristors with fundamental photonic building blocks used in both communication and computing applications. This allows a path towards realizing low-loss, non-volatile optical elements with near-zero static power consumption.

## Introduction

Over the past few decades, processor performance has scaled accordingly to Moore’s Law, however, there remains a fundamental limit in current computer architectures: the von-Neumann bottleneck^1,2^. This inherently places a limit on the amount of data that can be transferred from memory to processor. In 2008, Hewlett Packard Labs offered a potential solution towards non-volatile in-memory computing that can surpass the limitations of current von-Neumann designs^3,4^. These devices known as memristors exhibit hysteretic current-voltage (I-V) behavior which enables multi-bit non-volatile resistance states^5^. Memristors have thus emerged as a leading candidate for implementing analog based neuromorphic computing systems in the pursuit of mimicking/harnessing the behavior of mammalian brains^3,5–8^. These two-terminal devices allow a high degree of integration density in the form of nm-sized crossbar arrays, thus yielding energy-efficient and parallelized in-memory computing where data exchange between memory and a central processing unit is uninhibited^9–11^. More recently, there have been a few optical memristor demonstrations which fall under three fundamental mechanisms^12^: (1) the phase transitions^13–15^, (2) valency change^16–19^, and (3) electrochemical metallization^20^. The phase transition effect is due to the transformation of an insulating material into one with metallic properties and is driven by heat^21^. The valency effect involves oxygen vacancy formation in transition metal oxides (HfO_2_, Al_2_O_3_, TiO_2_, etc.) thus providing a conductive pathway between two electrodes^16–19,22^. The electrochemical metallization effect is based on the formation of a conductive filament composed of metal ions^20^. These three fundamental mechanisms can be further classified into two groups defined by their filamentary memristive opto-electronic functionality: (1) non-volatile phase shifters, (2) and non-volatile absorbers^20^. The work described here falls under non-volatile phase shifters where a number of electro-optical interactions happen and are not necessarily independent. For instance, the oxygen vacancy filamentation affects the optical refractive index via electrical conduction, yet charge traps can also occur^23–25^. In addition, the heat generated in nano-scale filamentary regions may morph amorphous transition metal oxides into polycrystalline or crystalline states^26^. Experimentally, it is difficult to separate these mechanisms, but experimental evidence in this paper suggests significant optical phase shifts occur through the formation of a conductive pathway via oxygen vacancies (VO^2+^). This conductive pathway has associated charge trap defects either within the HfO_2_, Al_2_O_3_ or at the interface of Al_2_O_3_/HfO_2_ during filamentation formation. These charge traps within the dielectric region effectively alter the built-in electric field and induce charges at the interface of the insulator/semiconductor region which in turn modulates the refractive index. Recent demonstrations of waveguide integrated optical memristive switches include ITO based latching switches^27^, ZnO based reflectors^16^, and Ag/a-Si/Si plasmonic absorbers^20^. Recently, we have leveraged our heterogeneous III-V/Si optical interconnect platform^28,29^ to integrate memristors based on semiconductor-insulator-semiconductor capacitors (SISCAP). This platform is suitable for complete device integration of quantum dot (QD) comb lasers^30–33^, III-V/Si SISCAP ring modulators^34–36^, Si-Ge avalanche photodetectors (APDs)^37–40^, QD APDs^28,41^, in-situ III-V/Si light monitors^42,43^, III-V/Si SISCAP optical filters^44,45^, and non-volatile phase shifters^17–19,22,46–50^, which are necessary for a fully integrated optical computing chip. These memristors are defined by the semiconductor-oxide interface and act as non-volatile phase shifters due to a multitude of effects described previously. The benefits of co-integrating silicon photonics and non-volatile memristors provides an attractive path towards eliminating the von-Neumann bottleneck. In addition, the memristive optical non-volatility allows post-fabrication error correction for phase sensitive silicon photonic devices while consuming zero power (supplementary note 1). As a result, we believe photonic memristors can contribute to energy efficient, non-volatile large scale integrated photonics such as: neuromorphic/brain inspired optical networks^51–60^, optical switching fabrics for tele/data-communications^61,62^, optical phase arrays^63,64^, quantum networks, and future optical computing architectures.

## Results

### III-V/Si SISCAP Memristors

**Fig. 1:**
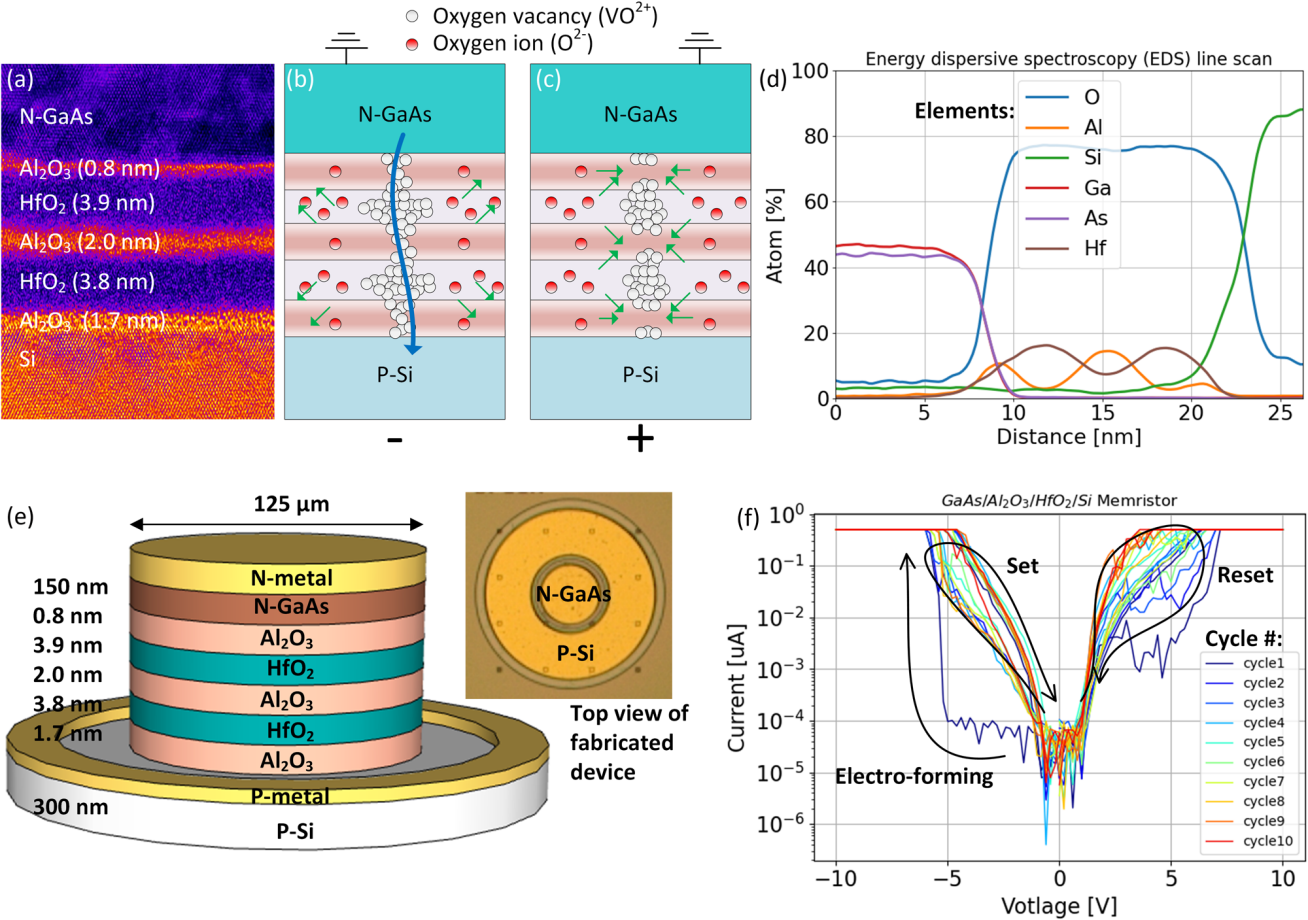
Description and characteristics of III-V/Si semiconductor-insulator-semiconductor capacitor (SISCAP). **a** HRTEM image of memristor stack, **b** “set” process: oxygen vacancy (VO^2+^) formation which initiates conductive filamentation, **c** “reset” process: break-up of filamentation, and (**d**) EDS line scan for atomic composition, **e** 3-D schematic of test structure, and **f** I-V curves of electro-forming, set, and reset processes.

The III-V/Si SISCAP memristor (Fig. 1a-e) is comprised of 300 nm thick p-type Si doped at 5 × 10^17 ^cm^−3^, alternating layers of HfO_2_/Al_2_O_3_, and 150 nm thick n-type GaAs doped at 3 × 10^18 ^cm^−3^. We chose a multi-layer HfO_2_/Al_2_O_3_ stack because Mahata et al., Khera et al. and Park et al., have shown improved resistive switching due to atomic inter-diffusion and promotion of oxygen vacancies (VO^2+^) at the HfO_2_/Al_2_O_3_ (HfAlO) interface^65–67^. Figure 1d shows the energy-dispersive X-ray spectroscopy (EDS) compositional mapping and indicates confirmation of the HfO_2_/Al_2_O_3_ stack as well as the HfAlO interface. Our previous attempts with pure Al_2_O_3_ yielded unstable and chaotic switching, therefore the inclusion of multi-layer HfO_2_/Al_2_O_3_ has helped. During the “set” process, VO^2+^ forms in both HfO_2_, Al_2_O_3_, and at the inter-diffused HfO_2_/Al_2_O_3_ (HfAlO) interface as shown in Fig. 1b and initiates a conductive path for electrons to flow. This turns the high impedance capacitor into a device exhibiting a low resistance state. During the “reset” process only the interfacial filament is believed to rupture first due to Al_2_O_3_ having less VO^2+^ than HfO_2_^65,66^, thus breaking the conductive path via a combination of Joule heating and field effect. This effectively restores the memristor in its high resistance state. We first evaluate the multi-layer memristor device electrically with a 125 μm wide capacitive structure shown in e. The voltage is first swept from 0 to −10 V with a compliance current = 0.5 μA which initiates the VO^2+^ electro-forming process as shown in f. Next, 10 voltage cycles were performed to examine the cyclability of the device with each cycle defined as: 0 V→ − 10 V → 0 V → 10 V →0 V. This allows us to observe multiple set/reset states. Given the large device surface area and random electro-formation^26,68^, consecutive set cycles were observed to increase the “set” current, (Fig. 1f) indicating increased filamentation sites. We did not further explore possible bias conditions to minimize this effect given limited test structures.

### III-V/Si Photonic SISCAP Memristors: Mach-Zehnder Interferometers (MZI)

**Fig. 2:**
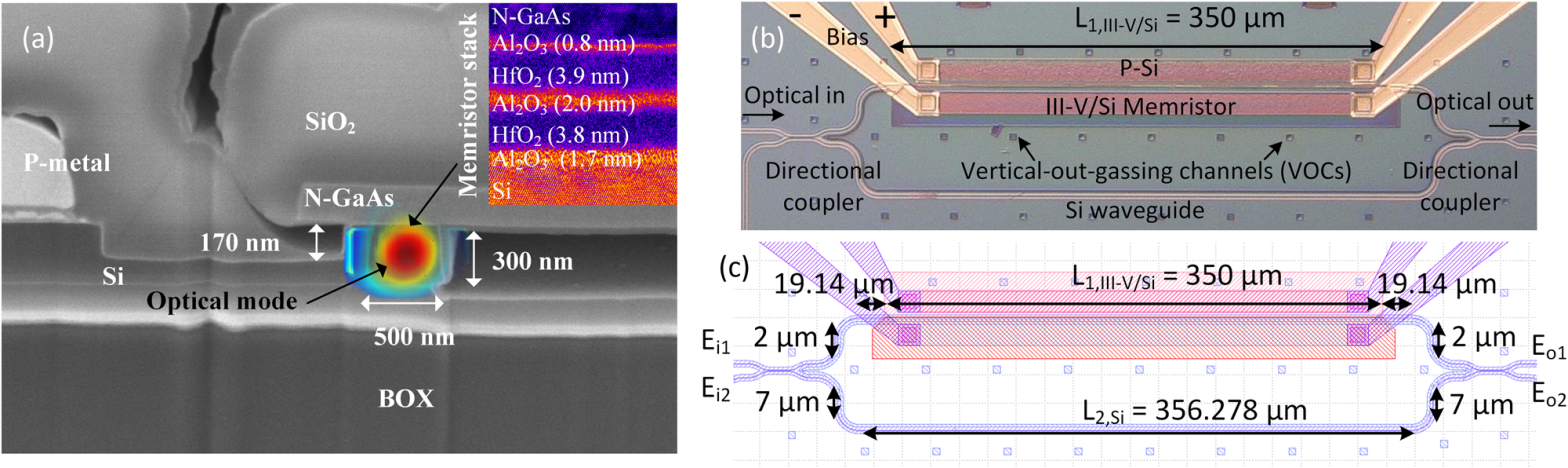
III-V/Si Mach-Zehnder memristor images and design dimensions. **a** SEM cross section with simulated guided optical mode. **b** Top view of fabricated device, and **c** device dimensions.

The optical waveguide of the III–V/Si MZI memristor is defined by a width, height, and etch depth of 500, 300, and 170 nm respectively as indicated in Fig. 2a. The Si is p-type doped at 5 × 10^17 ^cm^−3^ to ensure reasonable conductivity without affecting the optical loss significantly. Similar to the test capacitor in Fig. 1e, a multi-layer HfO_2_/Al_2_O_3_ stack sits on top of the silicon waveguide followed by a 150 nm-thick n-GaAs doped at 3 × 10^18 ^cm^−3^. Figure 2a shows the simulated transverse electric (TE) of the optical memristor. The inset shows a dielectric thicknesses of 1.7/3.8/2.0/3.9/0.8 nm for the Al_2_O_3_/HfO_2_/Al_2_O_3_/HfO_2_/Al_2_O_3_ memristor stack respectively. Assuming refractive indices of 1.75/1.90/1.75/1.90/1.75, the calculated optical confinement factors are Γ_Si_ = 64.49%, Γ_HfO2_ = 1.637%, and Γ_Al2O3_ = 0.82% with an overall effective index of n_eff_ = 3.176 and group index of n_g_ = 3.764. An Al_2_O_3_ layer is inserted in between the HfO_2_, because it was experimentally determined to be easier to wafer-bond Al_2_O_3_ to Al_2_O_3_ rather than HfO_2_. The choice of n-GaAs over p-GaAs was also two-fold: 1) lower optical absorption loss from dopants, and 2) easier III-V/Si laser integration. Also, GaAs exhibits ~ 4 × smaller electron effective mass and ~ 6 × larger electron mobility (m_e_^*^ = 0.063m_0_, μ_e_ = 8500 cm^2^/V-s) than crystalline Si (m_e_^*^ = 0.28m_0_, μ_e_ = 1400 cm^2^/V-s)^28,29,45^. Therefore, the plasma dispersion effect on index change in n-type GaAs is more efficient with lower free carrier absorption (FCA) loss.

The electric field transmission function of the top/bottom arm of the MZI (E_o1_, E_o2_) in Fig. 2c can be modeled with the following transfer matrix:

1 $$\left[\begin{array}{c}{E}_{o1}\\ {E}_{o2}\end{array}\right]=\left[\begin{array}{cc}t & jr\\ jr & t\end{array}\right]\left[\begin{array}{cc}{e}^{i{\phi }_{1}-{\zeta }_{1}} & 0\\ 0 & {e}^{i{\phi }_{2}-{\zeta }_{2}}\end{array}\right]\left[\begin{array}{cc}t & jr\\ jr & t\end{array}\right]\left[\begin{array}{c}{E}_{i1}\\ {E}_{i2}\end{array}\right]$$

2 $${I}_{o1}={|{E}_{o1}|}^{2},\,{I}_{o2}={|{E}_{o2}|}^{2}$$

3 $${\phi }_{1}=	 \, {\beta }_{1,si}{L}_{1,si}+{\beta }_{1,III-V/Si}{L}_{1,III-V/Si},\,{\phi }_{2}={\beta }_{2,si}{L}_{2,si}\\ {\zeta }_{1}=	 \, \frac{1}{2}{\alpha }_{1,si}{L}_{1,si}-\frac{1}{2}{\alpha }_{1,iii-v/si}{L}_{1,III-V/Si},\,{\zeta }_{2}=\frac{1}{2}{\alpha }_{2,si}{L}_{2,si}$$

**Fig. 3:**
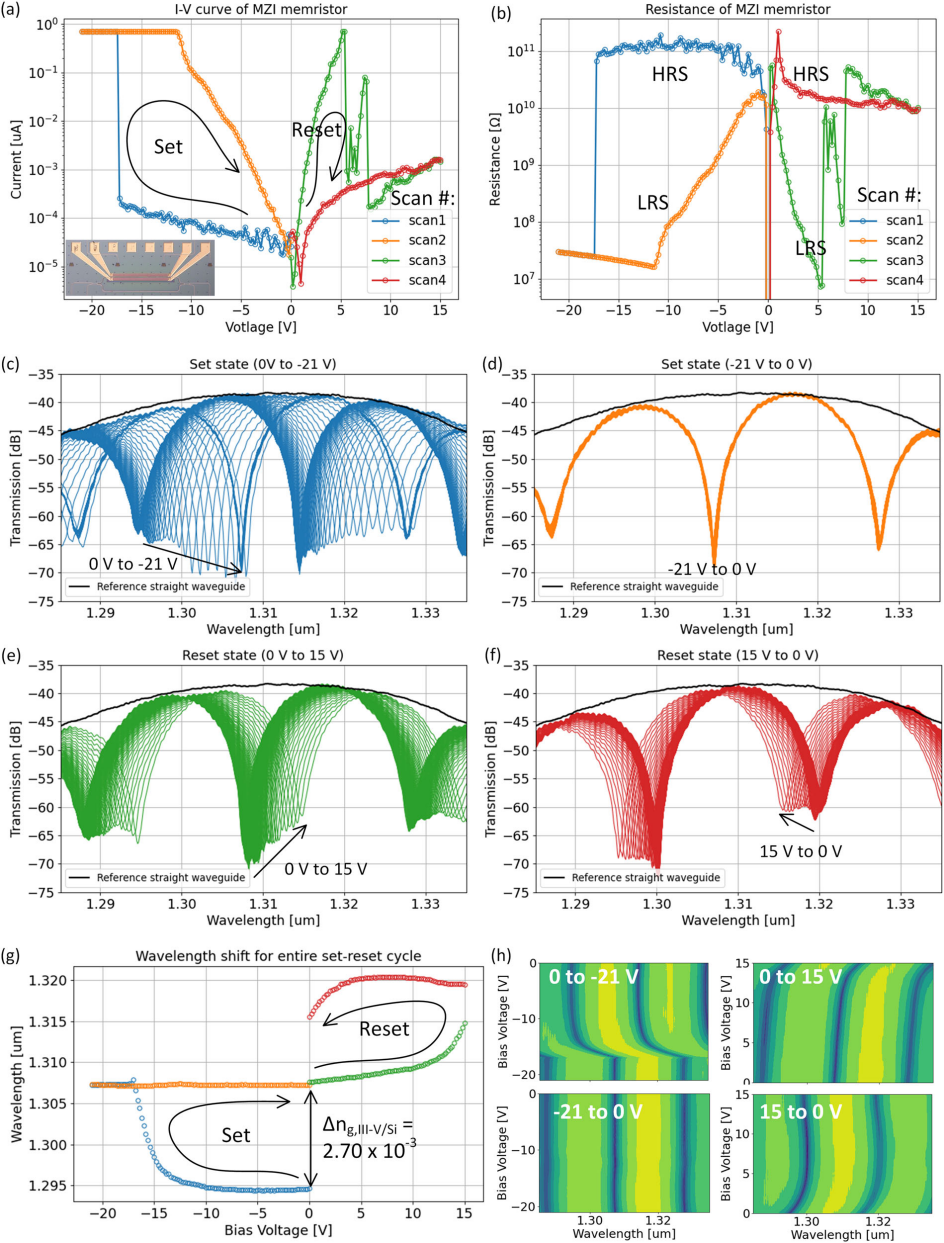
Electro-optical measurements of III-V/Si Mach-Zehnder memristor. **a** Measured I-V hysteresis indicating non-volatile memristive set/reset states. **b** Corresponding resistance indicating regions of high-resistance-states (HRS) and low-resistance-states (LRS). Measured optical spectrum for (**c**) “set” (0 to −21 V), **d** turning off “set” (−21 to 0 V), **e** “reset” (0 to 15 V), and **f** turning off “reset” (15 to 0 V). **g** Tracked resonance vs. voltage, **h** spectral evolution vs. voltage.

The through and cross port transmission of the directional couplers are defined by t and r respectively. The variables β_1,si_, β_2,si_, β_1,iii-v/si_, represent the propagation constants of the top arm silicon, bottom arm silicon, and top arm III-V/Si memristor waveguide respectively. α_1,si_, α_2,si_, α_1,iii-v/si_ represent the optical losses in the top arm, bottom arm, and top arm III-V memristor waveguide respectively. L_1,si_, L_2,si_, L_1,iii-v/si_ represent the corresponding lengths. Based on the transfer-matrix model, the two directional couplers have a power transfer coefficient of 49% assuming they are identical during fabrication. The measured spectrum indicates a free spectral range (FSR) of ~ 20.13 nm with an extinction ratio (ER) of ~ 31.1 dB near 1310 nm. The III–V/Si memristor region is located on the upper arm with a length of L_1,III-V/Si_ = 350 μm as shown in Fig. 2b. The p-doping is defined 2.0 μm away from the edge of the silicon waveguide and test structures indicated little to no effect on optical losses. The n-GaAs has a 200 nm overhang to the edge of the silicon waveguide such that III-V/Si bonding remains intact while avoiding contact with silicon pillars as shown in Fig. 2a. A fully etched deep trench is defined in between the MZI arms such that the p-Si is electrically isolated from the wafer-bonded n-GaAs region. The III-V/Si SISCAP structure operates as the memristor. In order to investigate non-volatile optical memory functionality, we first measure the current-voltage (I-V) relationship as shown in Fig. 3a. By voltage cycling from 0 → − 21 → 0 → 15 →0 V, a hysteresis curve is observed, therefore, confirming electrical memristor behavior. Figure 3b. illustrates the corresponding resistance indicating an initial high-resistance-state (HRS) which becomes a low-resistance-state (LRS) by applying a set voltage V_set_ = − 17.31 V.

By applying a reset voltage of V_reset_ > 5 V, a transition from the LRS to HRS can occur, thus concluding a reset back to the original electrical state. While taking I–V data, we simultaneously measured the optical spectral response with an optical spectrum analyzer (OSA). The optical response is shown in Fig. 3c–f and is color-coded according to the I–V curves in Fig. 3a–b. Applying a bias from 0 to −21 V results in a non-volatile wavelength shift of Δλ_non-volatile_ = 12.53 nm with near negligible optical losses (Fig. 3c). The wavelength shift is measured from the resonance dip (~1294.45 nm) where the arrow begins at 0 V and to the resonance dip (1306.98 nm) where the arrow ends with −21 V. Device insertion loss is discussed in supplementary note 5 and observed to be <0.5 dB. Ramping back down from −21 to 0 V does not shift the optical response back to the original state (Fig. 3d) and has a non-volatile wavelength stability of ~+/− 0.2 nm (35 GHz). This indicates a non-volatile phase shift of Δφ = 1.245π assuming a FSR = 20.13 nm, at essentially 0 power consumption (recorded current = 34 pA at 0 V). The group index (n_g_^III-V/Si^) of the III-V/Si memristor can be calculated by the following: FSR_MZI_ = λ^2^/(n_g_^lower^ L^lower^ − n_g_^upper^ L^upper^) where n_g_^lower^ L^lower^ - n_g_^upper^ L^upper^ = n_g_^Si^ L_Si_^lower^ – (n_g_^III-V/Si^ L^III-V/Si^ + n_g_^Si^ L_Si_^upper^). From this, the group index difference was calculated to be Δn_g_^III-V/Si^ = 2.70 × 10^−3^ which is quite significant. On separate devices, a full Δφ > 4π can be achieved with Δn_g_^III-V/Si^ = 13.7 × 10^−3^ (supplementary note 4). As we attempt to reset the device from 0 → + 15 → 0 V, a high resistance state is achieved. This shows we can electrically reset the device, albeit with the absence of an optical reset as shown in Fig. 3g, h. The disassociated coupling of electrical/optical reset may indicate the existence of residual defects from VO^2+^ formation (c) that can attributed to long-lived charge traps^69–72^. This is most likely the case since a Δn_g_^III-V/Si^ = 2.70 × 10^−3^ would require an untenable 20% change in the HfO_2_/Al_2_O_3_ multi-layer stack, assuming there are no thickness changes. We have attempted to image filamentation formation in the HfO_2_/Al_2_O_3_ multi-layer memristor, however, due to the reportedly small size (<5 nm) over a relatively large area of 0.5 μm × 350 μm, we were not able to find such an image. Experimentally, the filaments do exist due to the observation of I-V hysteresis shown in Fig. 3a. We believe the filament formation is not a single event since it would not explain the large phase shifts we observe. Instead, it is most likely a random collection of filaments that are formed along the entire 0.5 μm × 350 μm hybrid III-V/Si area with associated charge trap defects. The amount of charge trap defects is estimate to be > 9 × 10^19 ^cm^−3^ in order to explain the large group index changes and is simulated in supplementary note 1. To verify the absence of any significant material degradation, we performed HRTEM imaging for the initial, set, and reset states as shown in supplementary note 2. The initial state refers to a pristine, un-biased sample. Geometric phase analysis (GPA) was also performed to fully quantify any strain deformation that may occur for initial, set, and reset states. The results, shown in the supplementary note, indicates the set process contributes to an increase of nano-scaled oxide/semiconductor interfacial strains ranging from −0.5 to 0.5 % for the in-plane (E_xx_) and out-of-plane (E_yy_) directions. To the degree these nano-scaled strain points contribute to electron charge traps or VO^2+^ is quantitatively unknown in our device, but are known to exist in other studies^71,73–75^. In order to quantitatively assess the charge trap density needed to observe experimental phase shifts (Δn_g_^III-V/Si^ = 2.70 × 10^−3^), we employed SILVACO ATLAS. This is a two-dimensional solver capable of performing energy-band diagram and charge concentration calculations to theoretically predict optical effective and group index changes as a function of trapped charge density (Q_TC_). Based on the electron and hole concentrations, a spatial change in index can be calculated as^76,77^: Δn(x,y) (at 1310 nm) = − 6.2 × 10^−22^ ΔN(x,y) −6 × 10^−18^ ΔP(x,y)^0.8^, where x and y are the 2D lateral and vertical dimensions as detailed in supplementary note 1. Δn(x,y) is then used in an optical finite-difference-eigenmode (FDE) solver to calculate non-volatile group index changes Δn_g,non-volatile_ vs. Q_TC_ as indicated in supplementary note 1. A charge trap density of Q_TC_ = 9 × 10^19 ^cm^−3^, yields a group index change of ~ 1.75 × 10^−3^ which is not too far off from our experimentally determined value of Δn_g_^III-V/Si^ = 2.70 × 10^−3^. An extreme case of a phase shift Δφ > 4π is demonstrated in supplementary note 4 and exhibits an index change of Δn_g_^III-V/Si^ = 13.7 × 10^−3^ with essentially 0 static power consumption. This large change indicates residual charge trap densities > 9 × 10^19 ^cm^−3^. Set switching speeds were demonstrated to be ~ 1 ns^17^. In regards to device-to-device variability, we only had 2 devices to work with and based on this, there is indeed variability in terms of phase change. While it is difficult to assess the statistical significance of variability, we are currently fabricating much more memristive MZI devices with a foundry to determine so in the future.

**Fig. 4:**
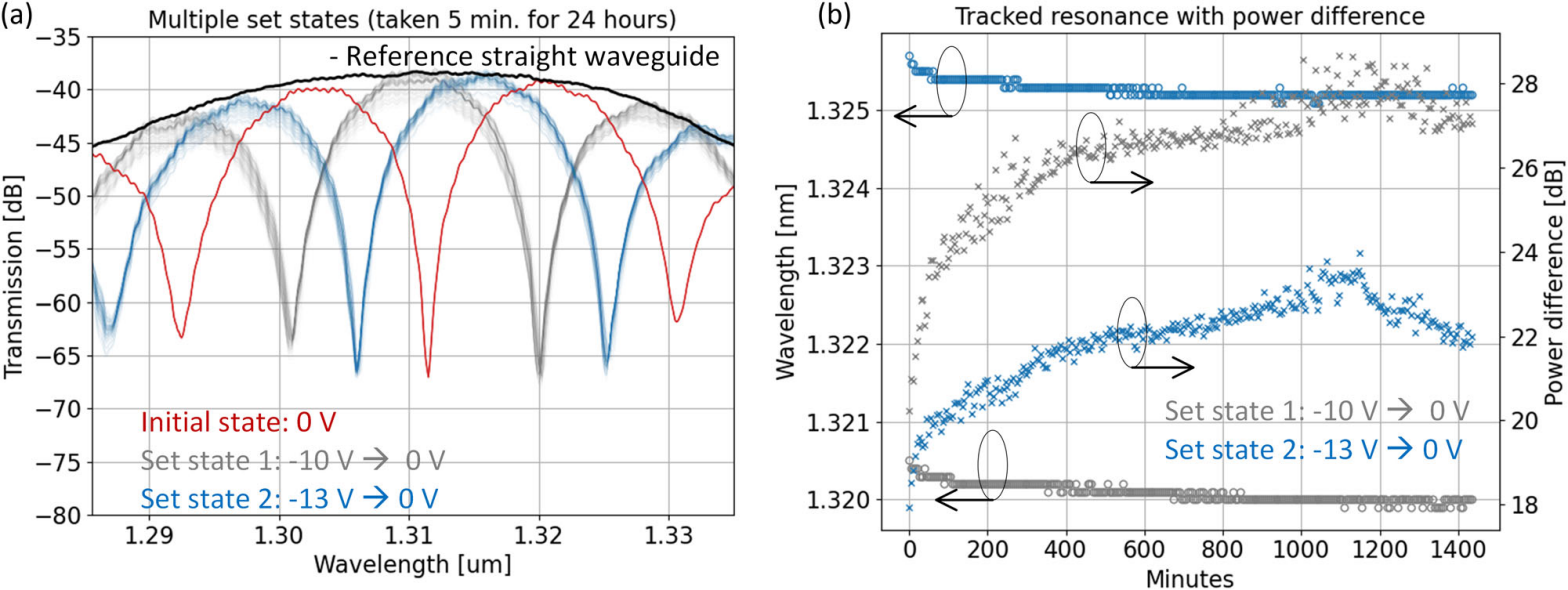
Time duration tests for multi-state photonic memristor. **a** 24 hour measured optical response taken every 5 minutes for (**a**) set state 1 (gray), set state 2 (blue), **b** tracking resonance wavelength and power difference of set state 1 and 2 over 24 hours.

In order to test the reliability of the non-volatile states, time duration tests were performed by biasing the MZI memristor into multiple non-volatile states and the optical response was recorded for 24 hours every 5 minutes. In Fig. 4a, the red curve is the initial state at 0 V. Next, we bias the device to “set state 1”, turn off the bias and record the optical output for 24 hours. Next, we perform the same procedure for “set state 2”. As observed in Fig. 4a, the optical response in the non-volatile set states are stable up to 24 hours and most likely beyond. In order to quantify this stability, we extracted the resonance dips over time (indicated by °). As a result, the multiple set states are stable by ~+/− 0.05 nm (8.77 GHz) within a 24 hour time frame indicating stable non-volatile behavior. The extracted non-volatile power difference between the initial state and different set states are indicated by ‘×’ in Fig. 4b and show the possibility of multi-bit non-volatile weighting.

We were able to perform 3 cycles from set to reset before the device failed. In each of these cycles, a non-volatile π phase shift was achieved. The measured optical spectrum and corresponding I–V curves are shown in supplementary note 6.

### III-V/Si Photonic SISCAP Memristors: (De-) Interleavers

Ring assisted asymmetric MZIs (RAMZIs) find use as filters for flat-top response with improved channel XT^28,36,44,45^. They also find use as linearized transfer functions for improved bit resolution in optical neural networks (ONNs)^78^ as well as RF photonics^79^. For the (de-)interleaver architecture, we chose a single ring resonator assisted asymmetric Mach-Zehnder interferometer (1-RAMZI) where the transmission passbands can be expressed as:

4 $${\varPhi }_{1-ring\, RAMZI}= 	\left[\begin{array}{cc}{c}_{1}(\lambda ) & -j{s}_{1}(\lambda )\\ -j{s}_{1}(\lambda ) & {c}_{1}(\lambda )\end{array}\right]\left[\begin{array}{cc}{A}^{R}(z)/A(z) & 0\\ 0 & {e}^{j2\pi {n}_{g}(\lambda ){L}_{ring}/\lambda }\end{array}\right]\\ 	 \left[\begin{array}{cc}{c}_{0}(\lambda ) & -j{s}_{0}(\lambda )\\ -j{s}_{0}(\lambda ) & {c}_{0}(\lambda )\end{array}\right]$$

5 $${A}^{R}(z)=\sqrt{1-{\kappa }_{r}}+{\left({e}^{j2\pi {n}_{g}(\lambda ){L}_{ring}/\lambda }\right)}^{-2},$$

6 $$A(z)=1+\sqrt{1-{\kappa }_{r}}{\left({e}^{j2\pi {n}_{g}(\lambda ){L}_{ring}/\lambda }\right)}^{-2}$$

The AMZI bar and cross port transmission are respectively defined similarly for the MZI filter with the addition that the κ_r_ is the ring coupling coefficient. The FSR is defined by the ring circumference such that the FSR = c/n_g_/L. Therefore, a channel spacing of 65 GHz for the 1-ring AMZI requires L_ring_ = 1200 μm for a calculated group index of n_g_ = 3.78. The ideal ring resonator coupling for a 1-RAMZI occurs at κ_r_ = 0.89. Details of this device under volatile SISCAP phase shift operation can be found in^28,36,44,45^.

**Fig. 5:**
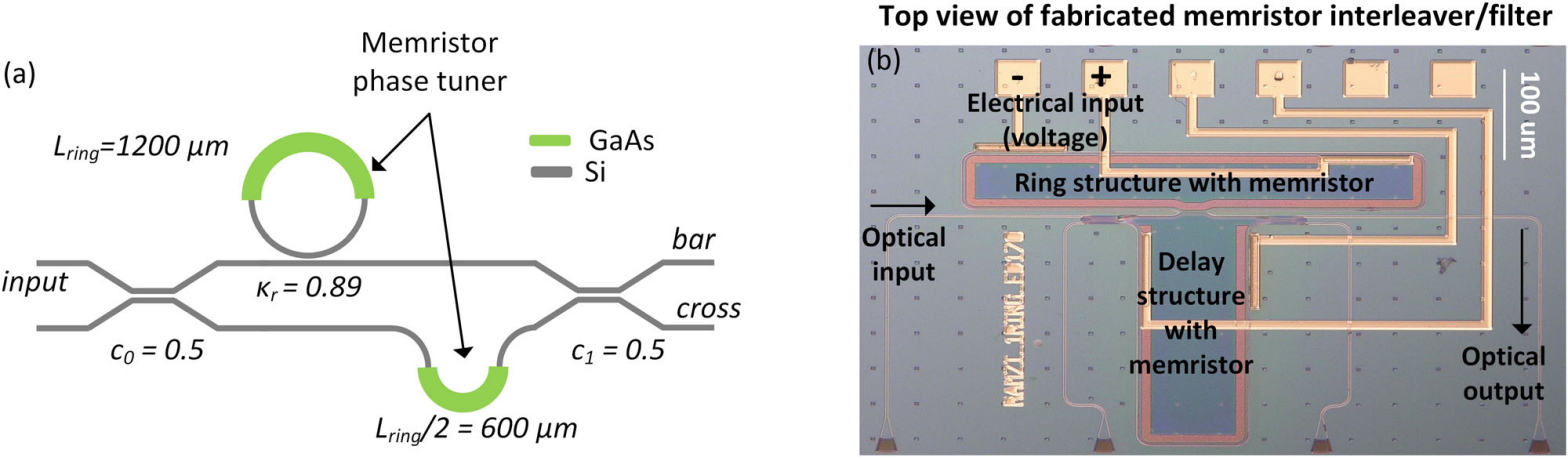
Design schematic and fabricated device of III-V/Si (de-) interleaver memristor. **a** Schematic of 65 GHz 1-ring assisted Mach-Zehnder interferometer (RAMZI) (de-)interleaver with memristive phase tuning elements (green), and **b** top view of fabricated device.

**Fig. 6:**
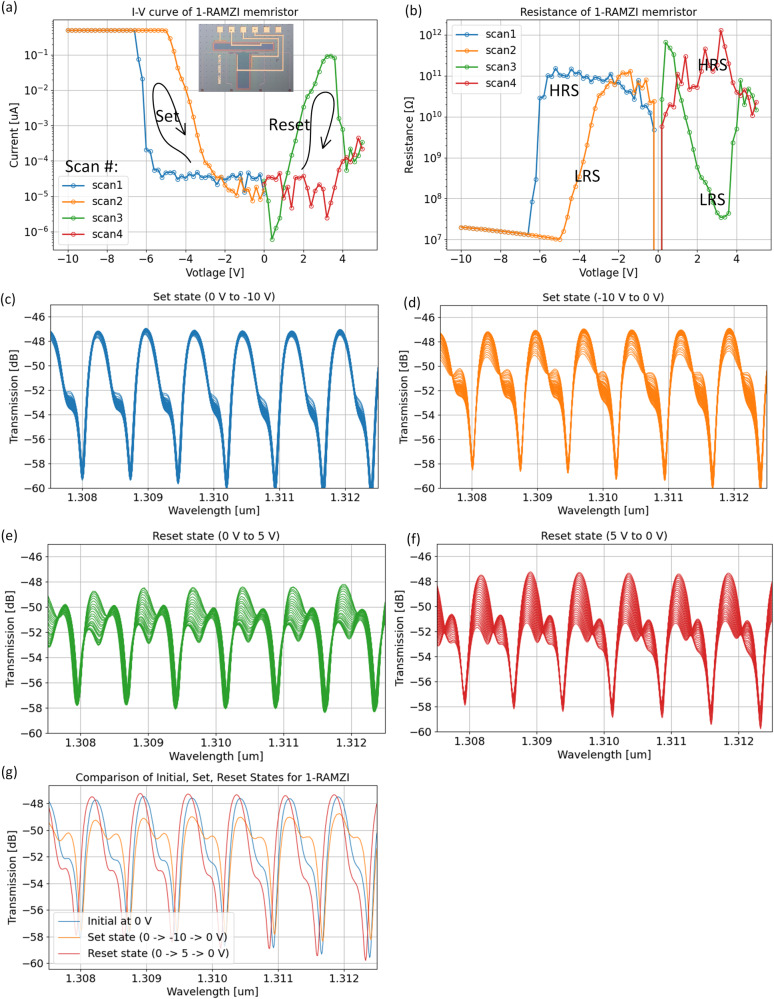
Electro-optical measurements of III-V/Si III-V/Si (de-) interleaver. **a** Measured I-V hysteresis indicating non-volatile memristive set/reset states and (**b**) corresponding resistance states. Measured optical spectrum for (**c**) “set state” (0 to −10 V), **d** turning off “set state” (−10 to 0 V), **e** “reset state” (0 to 5 V), and **f** turning off “reset state” (5 to 0 V). **g** Overlapped spectrum for final initial, set, and reset states.

The III–V/Si SISCAP structure on the ring or delay path can operate as the optical memristor as shown in Fig. 5a–b. In order to investigate non-volatile optical memory functionality, we once again measure the current-voltage (I–V) relationship as shown in Fig. 6a. A hysteresis curve is observed, therefore, confirming electrical memristor behavior along with non-volatile conductance (Fig. 6b). While taking I–V data, we simultaneously measured the spectral response with an OSA. The optical response is shown in Fig. 6c–f and is color-coded according to the I–V curves in Fig. 6a. Applying a bias from 0 to −10 V and back down to 0 V results in a non-volatile change in the passbands. In an attempt to reset the optical response, we apply a bias from 0 to 5 V and back down to 0 V. A passband shape similar to the initial one (Fig. 6g) is obtained with minor differences possibly associated with remaining VO^2+^ charge traps. Transfer matrix modeling from Eqs. (4)–(6) indicate a ring resonator phase difference of ~ 0.89π (Δn_g_^III-V/Si^ = 0.48 × 10^-3^) from the initial to set state. It is observed that the “set” voltage (−6 V) is much less than that of the MZI (−17 V) and may be due to the differences in memristor area by a factor of 3.4. The 1-RAMZI differs from the MZI in that a phase change on one of the tuning elements will significantly affect the passband shape. Large wavelength shifts would require the tuning of both the ring and delay arm shown in Fig. 5a. If these optical memristive filters were to be used as non-volatile elements, reliability and retention times would be of interest.

**Fig. 7:**
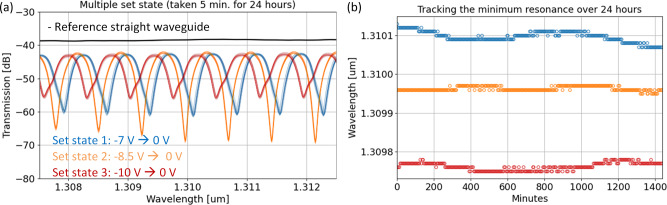
Time duration tests for multi-state, asymmetric Mach-Zehnder interferometer (AMZI) memristor. **a** Multiple set states for 3^rd^ order (de-)interleaver, and **b** non-volatile stability over a 24 hour period by tracking spectra minima.

We performed these time duration tests on a separate (de-)interleaver known as a 3^rd^ order AMZI. Multiple non-volatile set states were achieved with each one measured every 5 minutes for a 24 hour period. The overlapped filter shapes are shown in Fig. 7a. Three minima from each state were also tracked (Fig. 7b) and exhibited +/− 0.02 nm (3.51 GHz) change for the worst case (blue curve) for this 24 hour period.

## Discussion

There are many competing technologies that are capable of exhibiting optical non-volatility. Youngblood, et al. and Fang, et al. have surveyed a comprehensive list of performance metrics for the current state-of-the-art. Here we compare our work with some of the selected metrics from those papers^80,81^ as well as additional devices in Table 1. Magneto-optical switches with non-volatility have been demonstrated on single-crystalline cerium-substituted yttrium iron garnet (Ce:YIG) in both MZI and ring configuration with 1 MHz switching speeds^82^. A π phase shift with 25 dB ER was achieved for the Ce:YIG MZI albeit with 10 dB loss. Recently, there have been significant work on ferro-electric BaTiO_3_ with impressive non-volatile multi-states and cyclability^83^. However, the authors require a reset sequence consisting of thousands of pulses. A CMOS compatible non-volatile MZI was demonstrated with <20 pJ switching energy and 25 dB ER, although the switching speed remains to be improved^84^. Phase-change materials are a heavily researched topic^85–88^ and have recently been demonstrated to have excellent retention time of 77 days, cyclability reaching in the 1000 s, and 5 bit resolution, and require the need to change from amorphous to crystalline states^89^. Recently, an electrically driven memristive ring resonator with non-volatility was demonstrated on a III-V/Si hybrid platform with a switching and energy of <1 ns and 0.15 pJ^17^. Cyclability was shown to be 1000 with an insertion loss of 4 dB. In this work, we demonstrate a III-V/Si memristive MZI capable of a π phase shift with <0.5 dB insertion loss, 33 dB ER, 6 non-volatile states and non-volatile state retention lasting > 24 hours. However, 3 cycles were only demonstrated because of limited samples and device failure.

**Table 1:** Experimental demonstrations of non-volatile phase shifter elements

Metrics	Magneto-Optic (MZI)^82^	Ferro-electric BaTiO_3_ (ring)^83^	Charge-Trap (MZI)^84^	Phase Change Memory (MZI)^89^	Mem-Resonator (ring)^17^	Mem-MZI (this work) (MZI)
Switching speed	1 MHz	1.3 MHz	1.66 Hz	~ kHz	<1 ns	2 ms
Switching energy	100 nJ	4.6-26.7 pJ	<20 pJ	232 nJ	0.15 – 0.36 pJ	1500 nJ
Retention time	N/A	10 hours	N/A	77 days	12 hours	>24 hours
Bits/states	1 bit	> 3 bit	1 bit	5 bit	3 states	6 states
Footprint (μm^2^)	216,000	22,853	50,000	10,000	2513	28,000
ER (dB)	25	12	25	25	15	33
Phase shift	π	0.15π	π	π	0.18π	π
Insertion loss (dB)	10.0	>0.07	N/A	<1.0	4.0	<0.5
Cycles	>7	300	N/A	1600	1000	3
CMOS compatible?	×	o	✓	o	o	o

For CMOS compatibility, ‘×’ means no, ‘o’ means questionable, and ‘✓’ means yes.

**Fig. 8:**
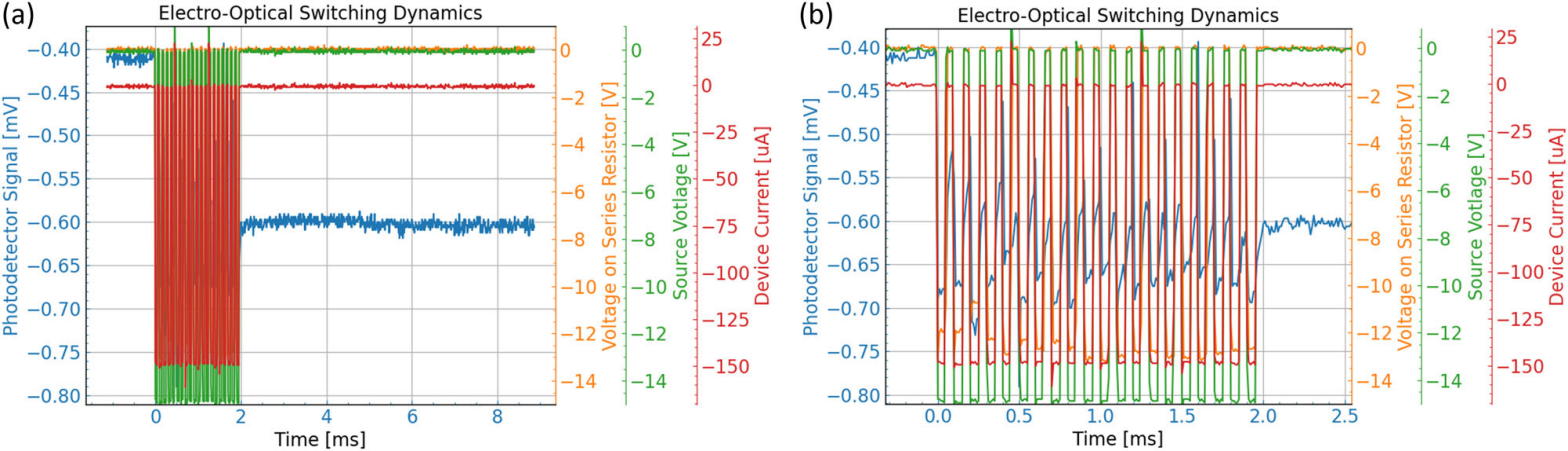
Non-volatile switching dynamics of III-V/Si memristor. **a** Time measurement of non-volatile switching behavior in the memristive MZI, **b** up-close image of electrical and optical dynamic quantities during switching process.

We evaluated non-volatile switching speed and energy by probing the device with high speed and voltages generated by a Keysight B1500A semiconductor parameter analyzer. A 100 kΩ resistor is placed in series with the device under test such that accurate device current can be measured by monitoring the voltage drop across the resistor. A non-volatile π phase shift was achieved by applying twenty 50 μs pulses with a 50% duty cycle and an amplitude of – 15 V as shown in Fig. 8. The total non-volatile switching time is ~2 milliseconds. Switching energy is calculated by using the measured device current (red curve), voltage (orange curve), the number of time pulses and yielded ~ 1500 nJ for a single non-volatile event. The photodetector signal (blue curve) shows a clear permanent shift in optical power after the voltage pulses are turned off at the end of the 2 millisecond sequence, thus indicating true zero static power consumption albeit with high 1500 nJ switching energies.

The switching dynamics of the optical and electrical signals shown in Fig. 8a, b uncovers some of the physical mechanisms occurring within the device. By observing the region of the first source voltage pulse (green), it is observed that a large change in optical amplitude happens in conjunction with increased device current due to heat. After the first source voltage pulse returns back to 0, no current is detected, however, there is a noticeable residual change in the photodetector signal possibly due to a number of effects discussed earlier in the manuscript. By sequentially initiating this mechanism, a non-volatile change in the optical signal can occur with near 0 power drawn inside the device. This indicates a heat mechanism is required to initiate the electrical and optical non-volatility.

## Conclusion

Over the past few decades, processor performance has scaled accordingly to Moore’s Law, however, there remains a fundamental limit in current computer architectures: the von-Neumann bottleneck. This inherently results in the need to transfer massive data between processor and memory with an intrinsic limit on bandwidth × distance plus increasing interconnect power consumption. As a major step towards breaking this bottleneck (especially for photonic neuromorphic computing), the work described here enables volatile operation for low-power, high-speed, on-chip training (supplementary note 3) and non-volatile memristive optical memory for inference (supplementary note 8). This is all done on a heterogeneous III–V/Si platform capable of integrating all the necessary components needed for next generation applications such as: neuromorphic/brain inspired optical networks^51–59,90^, optical switching fabrics for tele/data-communications^61,62^, optical phase arrays^63,64^, quantum networks, and future optical computing architectures. In particular, this work demonstrates for the first time, co-integration of III-V/Si memristors with optical MZI and (de-)interleaver filters which are key components in both communication and computing applications. The III-V/Si MZI memristor exhibits non-volatile optical phase shifts ~ π (Δn_g_^III-V/Si^ = 2.70 × 10^−3^) with ~ 33 dB extinction ratio while under 0 electrical power consumption, albeit with high switching energies of ~1500 nJ. We demonstrate 6 non-volatile states with each state capable of 4 Gbps modulation. The III-V/Si (de-)interleaver memristor were also demonstrated to exhibit memristive non-volatile passband transformation with full set/reset states for 1-RAMZI and 2^nd^ order AMZI architectures. Time duration tests were performed on all devices and indicated non-volatility up to 24 hours and most likely beyond. In addition, the memristive optical non-volatility allows for post-fabrication error correction of phase sensitive silicon photonic devices while consuming zero power as shown in the supplementary note 1.

## Methods

**Fig. 9:**
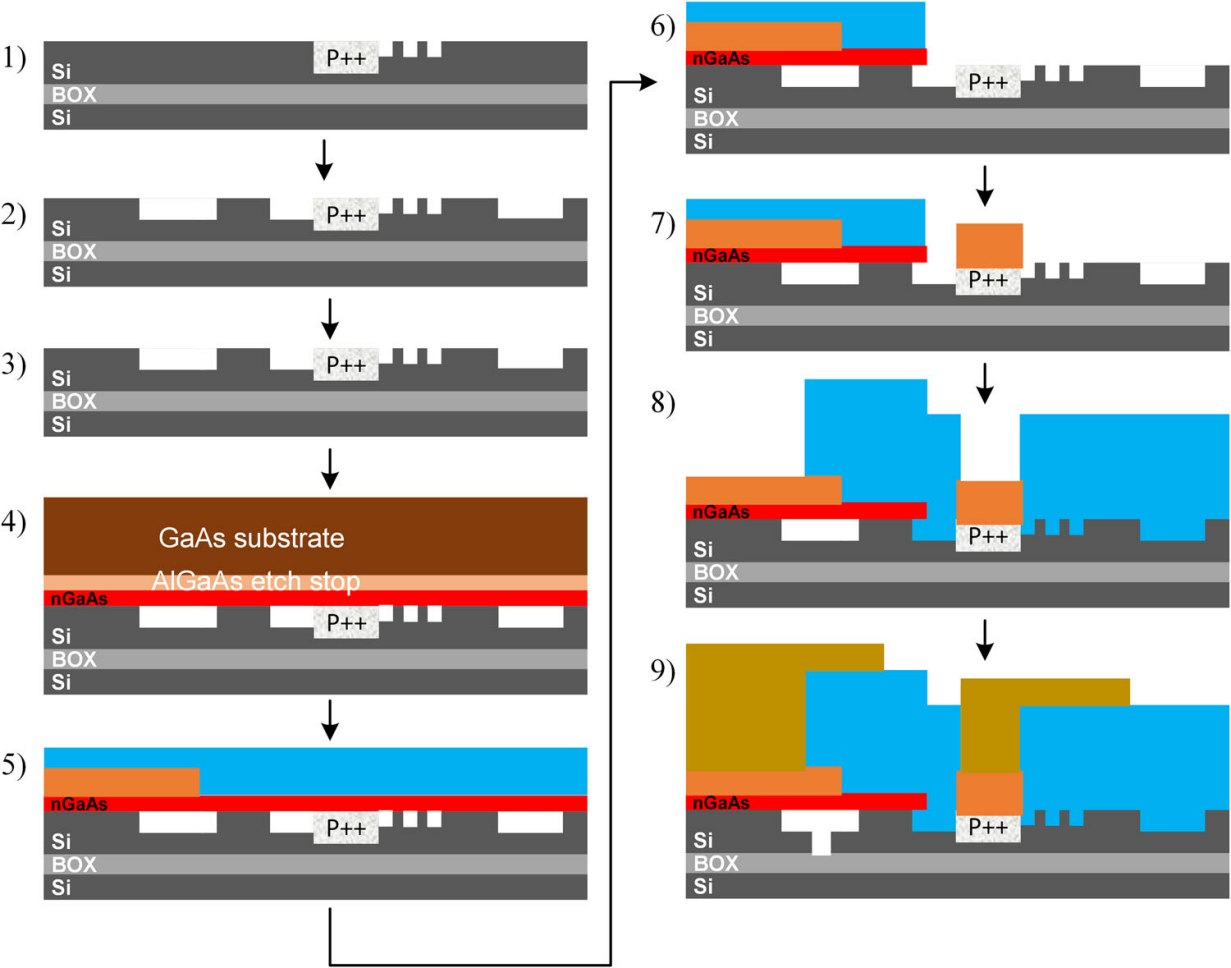
Fabrication flow of heterogeneous III-V/Si photonic memristor devices. 1) – 3) silicon processing, 4) wafer-bonding, 5) – 6) III-V processing.

**Fig. 10:**
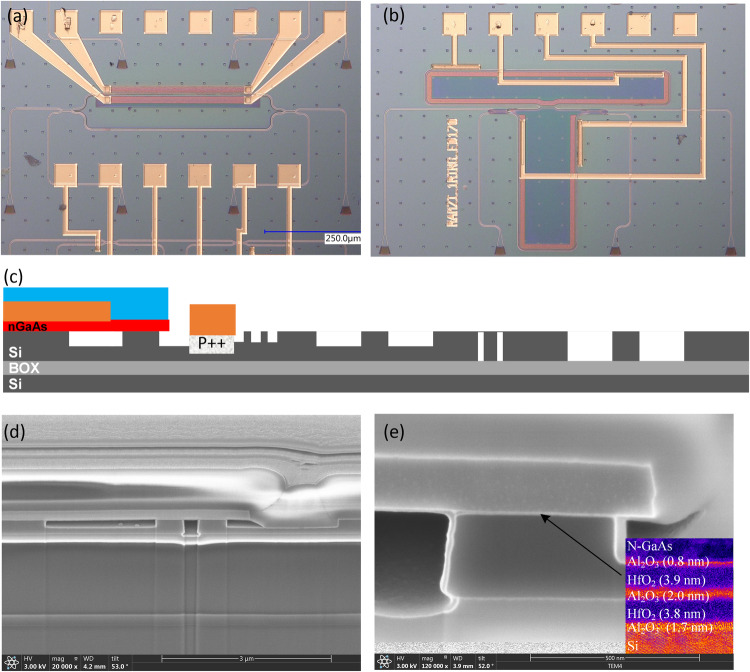
Microscope images of fabricated III-V/Si photonic memristor devices. **a** MZI, **b** 1-RAMZI filter. **c** Schematic of 2-D cross-section. **d**, **e** SEM image of cross-section.

The entire fabrication flow is shown in Fig. 9. Device fabrication begins with a SOI wafer consisting of a 350 nm top silicon layer and a 2 μm buried oxide (BOX) layer. Thermal oxidation is used to thin the top silicon down to 300 nm and buffered hydrofluoric (HF) acid etching is used to remove the oxide resulting in a pristine silicon surface. Silicon waveguides, grating couplers, and vertical out-gassing channels (VOCs) are all lithographically defined by a deep-UV (248 nm) ASML stepper. Silicon etching is performed with Cl_2_-based gas chemistry. The p ++ silicon contacts are formed via boron implantation. Next, the SOI wafer goes through a Piranha clean followed by buffered hydrofluoric (HF) acid etching to remove any organics and residual oxides. Next, an oxygen plasma clean is performed followed by a SC1 and SC2 clean. The III-V wafer is cleaned using acetone, methanol, and IPA, followed by O_2_ plasma cleaning and a 1 minute NH_4_OH:H_2_O (1:10) dip. Next a dielectric of Al_2_O_3_ is deposited onto both GaAs and SOI wafers via atomic layer deposition (ALD) by using 5 cycles of trimethylaluminum (TMA) + H_2_O at 300 °C with a target thickness of 0.5 nm on each side. Next, a thickness target of 3 nm HfO_2_ is deposited on each sample via 30 cycles of tetrakis (ethylmethylamino) hafnium (TEMAH) + H_2_O at 300 °C. Finally, a thickness target of 1 nm Al_2_O_3_ is deposited on each sample via 10 cycles of TMA + H_2_O. The two samples are then wafer-bonded under pressure for 250 °C (15 hours). Next, the backside of the III-V is mechanically thinned down to 100 μm. An Al_0.20_Ga_0.80_As etch stop layer allows selective removal of the remaining GaAs substrate via wet etching (H_2_O_2_:NH_4_OH (30:1)) as shown in Fig. 9 (step 4). The Al_0.20_Ga_0.80_As is finally removed in buffered hydrofluoric acid (HF), thus leaving a clean 150 nm-thick n-GaAs on silicon. The n-contact on n-GaAs consists of Ge/Au/Ni/Au/Pd/Ti (400/400/240/4000/200/200 Å). Metal contact with the p-Si consists of Ni/Ge/Au/Ni/Au/Ti (50/300/300/200/5000/200 Å). A plasma enhanced chemical vapor deposition (PECVD) SiO_2_ cladding is deposited and via holes are defined and etched. Ti/Au metal probe pads are finally defined to make contact with n-GaAs and p-Si layers. Figure 10a, b shows images of the devices measured in this manuscript. Figure 10c–e shows a schematic of the cross section as well as the associated SEM images.

The experimental setup for measuring non-volatile switching speeds are detailed in supplementary note 7.

### Supplementary information

Supplementary Information

## Data Availability

The data that support the findings of this study are available from the corresponding author on reasonable request.

## References

[1] Beyond von Neumann. *Nat. Nanotechnol*. **15**, 507–507 (2020). 10.1038/s41565-020-0738-x 32647166

[2] Sebastian A, Le Gallo M, Khaddam-Aljameh R, Eleftheriou E (2020). Memory devices and applications for in-memory computing. Nat. Nanotechnol..

[3] Strukov DB, Snider GS, Stewart DR, Williams RS (2008). The missing memristor found. Nature.

[4] Williams RS (2008). How we found the missing memristor. IEEE Spectrum.

[5] Rao M (2023). Thousands of conductance levels in memristors integrated on CMOS. Nature.

[6] Park S-O (2022). Experimental demonstration of highly reliable dynamic memristor for artificial neuron and neuromorphic computing. Nat. Commun..

[7] Vaughan O (2023). A history of memristors in five covers. Nat. Electron..

[8] Yao P (2020). Fully hardware-implemented memristor convolutional neural network. Nature.

[9] Li C (2017). Analogue signal and image processing with large memristor crossbars. Nat. Electron..

[10] Li C (2020). Analog content-addressable memories with memristors. Nat. Commun..

[11] Pedretti G (2021). Tree-based machine learning performed in-memory with memristive analog CAM. Nat. Commun..

[12] Koch U, Hoessbacher C, Emboras A, Leuthold J (2017). Optical memristive switches. J Electroceram.

[13] Kwon KC, Baek JH, Hong K, Kim SY, Jang HW (2022). Memristive devices based on two-dimensional transition metal chalcogenides for neuromorphic computing. Nano-Micro Lett..

[14] Yoshida M, Suzuki R, Zhang Y, Nakano M, Iwasa Y (2015). Memristive phase switching in two-dimensional 1T-TaS2 crystals. Sci. Adv..

[15] Li Y (2013). Ultrafast synaptic events in a chalcogenide memristor. Sci Rep.

[16] Battal E, Ozcan A, Okyay AK (2014). Resistive switching-based electro-optical modulation. Adv. Opt. Mater,.

[17] Tossoun B (2024). High-speed and energy-efficient non-volatile silicon photonic memory based on heterogeneously integrated memresonator. Nat. Commun..

[18] Fang Z (2023). Fast and energy-efficient non-volatile III-V-on-Silicon photonic phase shifter based on memristors. Adv. Opt. Mater,.

[19] Fang, Z. et al. High-speed and energy-efficient non-volatile memristive III- V-on-silicon photonic phase shifter. in *Optical Fiber Communication Conference (OFC)* paper W3G.3 (San Diego, California, 2023).

[20] Emboras A (2013). Nanoscale plasmonic memristor with optical readout functionality. Nano Lett..

[21] Joushaghani A (2015). Wavelength-size hybrid Si-VO2 waveguide electroabsorption optical switches and photodetectors. Opt. Express.

[22] Cheung, S. et al. Heterogeneous III-V/Si (De-)Interleaver Filters with Non-Volatile Memristive Behavior. in *2022 IEEE Photonics Conference (IPC)* 1–2 (2022). 10.1109/IPC53466.2022.9975647.

[23] Oh S-M, You H, Kim K-S, Lee Y-H, Cho W-J (2010). Electrical properties of HfO2 charge trap flash memory with SiO2/HfO2/Al2O3 engineered tunnel layer. Current Applied Physics.

[24] Cerbu F (2016). Intrinsic electron traps in atomic-layer deposited HfO2 insulators. Appl. Phys. Lett..

[25] Cheung, S. et al. Non-volatile heterogeneous III-V/Si photonics via optical charge-trap memory. *arXiv preprint arXiv:2305.17578* (2023).

[26] Zhang Y (2021). Evolution of the conductive filament system in HfO2-based memristors observed by direct atomic-scale imaging. Nat. Commun..

[27] Hoessbacher C (2014). The plasmonic memristor: a latching optical switch. Optica.

[28] Liang D (2022). An energy-efficient and bandwidth-scalable DWDM heterogeneous silicon photonics integration platform. IEEE J. Sel. Top. Quant. Electron..

[29] Liang, D. et al. Integrated Green DWDM photonics for next-gen high-performance computing. in *2020 Optical Fiber Communications Conference and Exhibition (OFC)* 1–3 (2020).

[30] Kurczveil, G., Descos, A., Liang, D., Fiorentino, M. & Beausoleil, R. Hybrid silicon quantum dot comb laser with record wide comb width. in *Frontiers in Optics paper FTu6E.6*. (OSA Technical Digest (Optica Publishing Group, 2020).

[31] Kurczveil, G. et al. On-Chip Hybrid silicon quantum dot comb laser with 14 error-free channels. in *2018 IEEE International Semiconductor Laser Conference (ISLC)* 1–2 (2018). 10.1109/ISLC.2018.8516175.

[32] Kurczveil G, Seyedi MA, Liang D, Fiorentino M, Beausoleil RG (2018). Error-free operation in a hybrid-silicon quantum dot comb laser. IEEE Photonics Technol. Lett..

[33] Kurczveil, G. et al. High-temperature error-free operation in a heterogeneous silicon quantum dot comb laser. in *2022 Optical Fiber Communications Conference and Exhibition (OFC)* 1–3 (San Diego, California, 2022).

[34] Srinivasan, S., Liang, D. & Beausoleil, R. G. High Temperature Performance of Heterogeneous MOSCAP Microring Modulators. in *2021 Optical Fiber Communications Conference and Exhibition (OFC)* 1–3 (San Francisco, CA, USA, 2021).

[35] Srinivasan, S., Liang, D. & Beausoleil, R. G. Heterogeneous SISCAP Microring Modulator for High-Speed Optical Communication. in *2020 European Conference on Optical Communications (ECOC)* 1–3 (2020). 10.1109/ECOC48923.2020.9333221.

[36] Cheung S (2022). Demonstration of a 17 × 25 Gb/s heterogeneous III-V/Si DWDM transmitter based on (De-) interleaved quantum dot optical frequency combs. J. Lightw. Technol..

[37] Yuan Y (2022). High responsivity Si-Ge waveguide avalanche photodiodes enhanced by loop reflector. IEEE J. Sel.Top. Quantum Electron..

[38] Yuan Y (2020). 64 Gbps PAM4 Si-Ge waveguide avalanche photodiodes with excellent temperature. Stability. J. Lightwave Technol..

[39] Yuan Y (2022). OSNR sensitivity analysis for Si-Ge avalanche photodiodes. IEEE Photonics Technology Letters.

[40] Huang Z (2016). 25 Gbps low-voltage waveguide Si–Ge avalanche photodiode. Optica.

[41] Tossoun B (2021). 32 Gbps heterogeneously integrated quantum dot waveguide avalanche photodiodes on silicon. Opt. Lett..

[42] Srinivasan, S., Liang, D. & Beausoleil, R. In-situ light measurement in heterogeneous gain media. in *2021 27th International Semiconductor Laser Conference (ISLC)* 1–2 (2021). 10.1109/ISLC51662.2021.9615660.

[43] Srinivasan, S., Liang, D. & Beausoleil, R. Non-invasive light monitoring for heterogeneous photonic integrated circuits. in *2021 IEEE Photonics Conference (IPC)* 1–2 (2021). 10.1109/IPC48725.2021.9593047.

[44] Cheung, S. et al. Ultra-Power Efficient Heterogeneous III-V/Si De-Interleavers for DWDM Optical Links. in *IEEE 17th International Conference on Group IV Photonics (GFP)* 1–2 (2021). 10.1109/GFP51802.2021.9673963.

[45] Cheung S (2022). Ultra-power-efficient heterogeneous III–V/Si MOSCAP (de-)interleavers for DWDM optical links. Photonics Res..

[46] Cheung, S. et al. Heterogeneous III-V/Si Non-Volatile Optical Memory: A Mach-Zehnder Memristor. in *2022 Conference on Lasers and Electro-Optics (CLEO)* 1–2 (San Jose, CA, 2022).

[47] Tossoun, B., Sheng, X., Paul Strachan, J., Liang, D. & Beausoleil, R. G. Hybrid silicon MOS optoelectronic memristor with non-volatile memory. in *2020 IEEE Photonics Conference (IPC)* 1–2 (2020). 10.1109/IPC47351.2020.9252481.

[48] Tossoun, B., Sheng, X., Strachan, J. P., Liang, D. & Beausoleil, R. G. Memristor Photonics. in *Photonics in Switching and Computing 2021* 1–2 (2021).

[49] Tossoun, B., Sheng, X., Strachan, J. P. & Liang, D. The Memristor Laser. in *2020 IEEE International Electron Devices Meeting (IEDM)* 7.6.1-7.6.4 (San Francisco, CA, USA, 2020).

[50] Cheung, S. et al. Non-Volatile Memristive III-V/Si Photonics. in *2023 IEEE Silicon Photonics Conference (SiPhotonics)* 1–2 (2023). 10.1109/SiPhotonics55903.2023.10141937.

[51] Shen Y (2017). Deep learning with coherent nanophotonic circuits. Nat. Photon..

[52] Xiao X, On MB, Vaerenbergh TV (2021). Large-scale and energy-efficient tensorized optical neural networks on III–V-on-silicon MOSCAP platform. APL Photonics.

[53] Peng H-T, Nahmias MA, de Lima TF, Tait AN, Shastri BJ (2018). Neuromorphic photonic integrated circuits. IEEE J. Sel. Top. Quantum Electron..

[54] Alexoudi T, Kanellos GT, Pleros N (2020). Optical RAM and integrated optical memories: a survey. Light Sci. Appl..

[55] Lian C (2022). Photonic (computational) memories: tunable nanophotonics for data storage and computing. Nanophotonics.

[56] Gu J (2022). ICCAD: G: Light in artificial intelligence: efficient neurocomputing with optical neural networks. IEEE Trans. Circuits Syst. II: Express Briefs.

[57] Ríos C (2019). In-memory computing on a photonic platform. Sci. Adv..

[58] Shastri BJ (2021). Photonics for artificial intelligence and neuromorphic computing. Nat. Photonics.

[59] Harris, N. C. et al. Programmable Nanophotonics for Quantum Information Processing and Artificial Intelligence. in *2022 27th OptoElectronics and Communications Conference (OECC) and 2022 International Conference on Photonics in Switching and Computing (PSC)* (Toyama, Japan, 2022). 10.23919/OECC/PSC53152.2022.9849929.

[60] El Srouji L (2022). Photonic and optoelectronic neuromorphic computing. APL Photonics.

[61] Yu R (2013). A scalable silicon photonic chip-scale optical switch for high performance computing systems. Opt. Express.

[62] Grani P, Proietti R, Cheung S, Ben Yoo SJ (2016). Flat-topology high-throughput compute node with AWGR-based optical-interconnects. J. Lightw. Technol..

[63] Sun J, Timurdogan E, Yaacobi A, Hosseini ES, Watts MR (2013). Large-scale nanophotonic phased array. Nature.

[64] Poulton CV (2019). Long-range LiDAR and free-space data communication with high-performance optical phased arrays. IEEE J. Select. Topics Quantum Electron.

[65] Khera EA (2022). Improved resistive switching characteristics of a multi-stacked HfO_2_/Al_2_O_3_/HfO_2_ RRAM structure for neuromorphic and synaptic applications: experimental and computational study. RSC Adv..

[66] Park S (2022). Multilayer redox-based HfOx/Al_2_O_3_/TiO_2_ memristive structures for neuromorphic computing. Sci. Rep..

[67] Mahata C, Kang M, Kim S (2020). Multi-level analog resistive switching characteristics in tri-layer HfO_2_/Al_2_O_3_/HfO_2_ based memristor on ITO electrode. Nanomaterials.

[68] Yang Y (2012). Observation of conducting filament growth in nanoscale resistive memories. Nat. Commun..

[69] Zahari F (2023). Trap-assisted memristive switching in HfO_2_-based devices studied by in situ soft and hard X-ray photoelectron spectroscopy. Adv. Electron. Mater..

[70] Zeumault A (2021). TCAD modeling of resistive-switching of HfO_2_ memristors: efficient device-circuit co-design for neuromorphic systems. Front. Nanotechnol..

[71] Liu Y-Y (2019). Characterizing the charge trapping across crystalline and amorphous Si/SiO_2_/HfO_2_ stacks from first-principle calculations. Phys. Rev. Appl..

[72] Lan X (2013). The interface inter-diffusion induced enhancement of the charge-trapping capability in HfO_2_/Al_2_O_3_ multilayered memory devices. Appl. Phys. Lett..

[73] Zou X (2012). Charge trapping-detrapping induced resistive switching in Ba0.7Sr0.3TiO3. AIP Adv..

[74] Xu Z (2022). Cationic interstitials: An overlooked ionic defect in memristors. Front. Chem..

[75] Banerjee W, Liu Q, Hwang H (2020). Engineering of defects in resistive random access memory devices. J. Appl. Phys..

[76] Chrostowski, L. & Hochberg, M. *Silicon Photonics Design: From Devices to Systems*. (Cambridge: Cambridge University Press, 2015).

[77] Reed, T., Mashanovich, G., Gardes, Y. & Thomson, J. Silicon optical modulators. *Nat. Photonics* **4**, 518–526 (2010).

[78] Yuan Y (2023). Low-phase quantization error Mach–Zehnder interferometers for high-precision optical neural network training. APL Photonics.

[79] Cardenas J (2013). Linearized silicon modulator based on a ring assisted Mach Zehnder inteferometer. Opt. Express.

[80] Youngblood N (2023). Integrated optical memristors. Nat. Photonics.

[81] Fang Z (2023). Non-volatile materials for programmable photonics. APL Mater..

[82] Murai T, Shoji Y, Nishiyama N, Mizumoto T (2020). Nonvolatile magneto-optical switches integrated with a magnet stripe array. Opt. Express.

[83] Geler-Kremer J (2022). A ferroelectric multilevel non-volatile photonic phase shifter. Nat. Photonics.

[84] Song J-F (2016). Integrated photonics with programmable non-volatile memory. Sci. Rep..

[85] Zheng J (2020). Nonvolatile electrically reconfigurable integrated photonic switch enabled by a silicon PIN diode heater. Adv. Mater..

[86] Zhang C (2023). Nonvolatile multilevel switching of silicon photonic devices with In_2_O_3_/GST segmented structures. Adv. Opt. Mater,.

[87] Ríos C (2022). Ultra-compact nonvolatile phase shifter based on electrically reprogrammable transparent phase change materials. PhotoniX.

[88] Zhou W (2023). In-memory photonic dot-product engine with electrically programmable weight banks. Nat. Commun..

[89] Chen R (2023). Non-volatile electrically programmable integrated photonics with a 5-bit operation. Nat. Commun..

[90] Srouji LE (2022). Tutorial: Photonic and optoelectronic neuromorphic computing. APL Photonics.

