# Supplementary material for: Energy efficient photonic memory based on electrically programmable embedded III-V/Si memristors: switches and filters
Source: Commun Eng. 2024 Mar 18;3:49. doi: 10.1038/s44172-024-00197-1 (PMC10956075; doi:10.1038/s44172-024-00197-1)
Supplement: Supplementary file 1 — Supplementary Information [file 44172_2024_197_MOESM1_ESM.pdf]

Supplementary

Stanley Cheung, Bassem Tossoun, Yuan Yuan, Yiwei Peng, Yingtao Hu, Wayne V. Sorin, Geza Kurczveil, Di Liang, and Raymond G. Beausoleil

Hewlett Packard Enterprise, Large-Scale Integrated Photonics Lab, Milpitas, CA. 95035, USA
[*]stanley.cheung@hpe.com, [*]bassem.tossoun@hpe.com

Supplementary Note 1:

Electro-Optical Simulations and Design

The effects of charge trap defects as a result of non-volatile III-V/Si memristive behavior was investigated using a 2-D electrical solver (SILVACO ATLAS [1]). Supplementary Table *1* lists the semiconductor material parameters used for optical and electronic TCAD simulations in this manuscript.

Supplementary Table 1: Material parameters used in electro-optical simulations

Material	E_g (eV)	χ (eV)	VBO (eV)	CBO (eV)	n	N_{TC} (cm^{-3})	φ_d (eV)
Al_2O_3	7.0 [2]	1.5 [3–5]	3.2 [2]	2.7 [2]	1.75	-	-
HfO_2	5.7 [2]	2.5 [2]	2.7 [2]	1.9 [2]	1.9	10^{19} - 10^{20} [6–8]	2.0 [9–12]
SiO_2	8.9 [2]	1.3 [2]	4.5 [2]	3.3 [2]	1.44	-	-
Si	1.1 [2]	4.1 [2]	-	-	3.507 [13]	-	-
GaAs	1.43 [1]	4.07 [1]	-	-	3.406 [13]	-	-

E_g: Energy gap, χ: electron affinity, n: refractive index (at $\lambda = 1310$ nm), VBO: valance band offset, CBO: conduction band offset, N_{TC}: trap density, φ_d: electron trap level

The electron/hole concentrations (Supplementary Fig. 1a), are simulated for various electrical biases. These simulated electron/hole spatial profiles ($\Delta P(x,y)$ and $\Delta N(x,y)$) can then be used to calculate a spatial refractive index change $\Delta n(x,y)$ at a wavelength of $\lambda = 1310$ nm by the following [14,15]:

$$\Delta n(x,y) = -6.2\times10^{-22}\,\Delta N(x,y) - 6.0\times10^{-18}\,\Delta P(x,y) \qquad (1)$$

This spatial refractive index change $\Delta n(x,y)$ is then exported into a 2D-FDE optical mode solver (Lumerical) and the non-volatile change in effective index ($\Delta_{neff,non\text{-}volatile}$) is calculated. Supplementary Fig. 1 shows the results of this procedure to determine $\Delta_{neff,non\text{-}volatile}$.

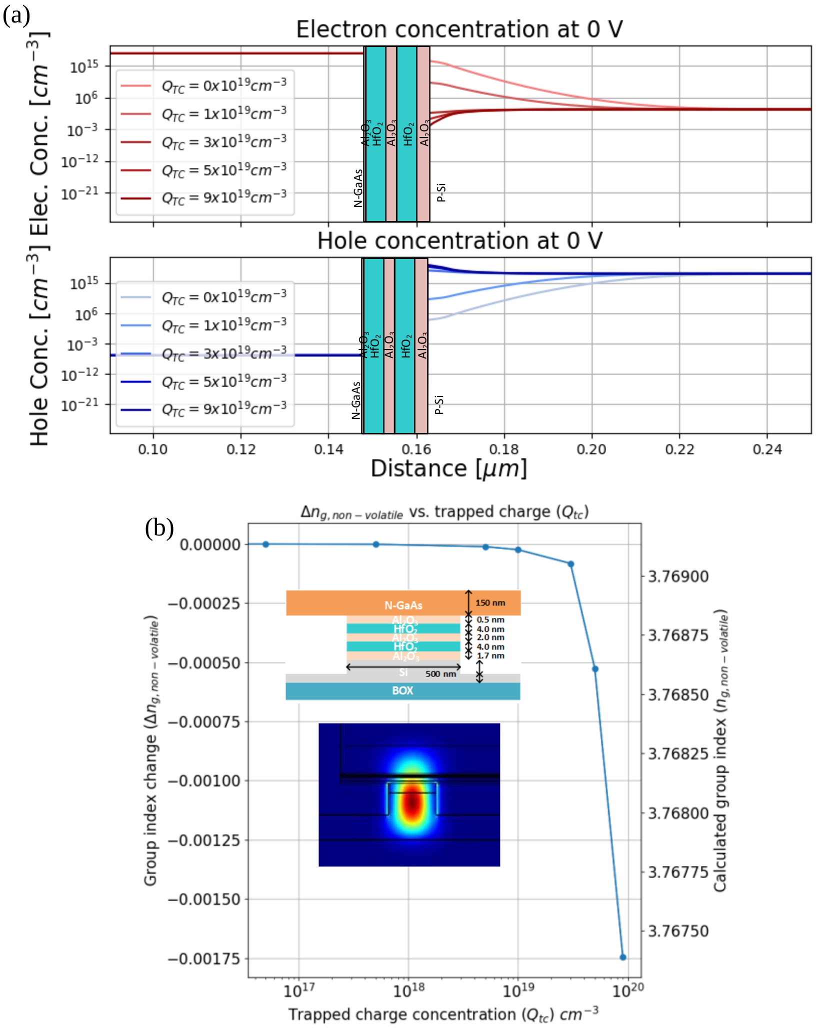

Supplementary Fig. 1. Electron and hole concentrations for various Q_{TC} after set process (0 → -15 → 0 V) and (b) non-volatile group index change $\Delta n_{g,,non\text{-}volatile}$ vs. Q_{TC}.

Post-Fabrication Trimming (0 power phase error correction)

The "set-and-forget" nature of the non-volatile III-V/Si memristor can be useful for post-fabrication trimming which leads to permanent phase error correction with virtually 0 static power consumption. This is quite important since phase error correction on high index contrast silicon photonics platforms employ micro-heaters that may consume several 10s of mW. Depletion or carrier injection devices can be used, but the phase shifts are nowhere as large as the results reported in this manuscript. As a result, we can correct for phase errors due to variations in waveguide width, thickness, and refractive index non-homogeneity. This has direct impact on phase sensitive devices/systems such as optical neural networks (ONN), arrayed waveguide gratings (AWGs), lattice filters, and (de-)interleavers. Dimensional changes on the resonant wavelength can be described by the following equation [16]:

$$\Delta\lambda_0 = \left(\lambda_0 / n_g\right)\sqrt{\left(dn_{eff} / dw \cdot \Delta w\right)^2 + \left(dn_{eff} / dt \cdot \Delta t\right)^2} \qquad (12)$$

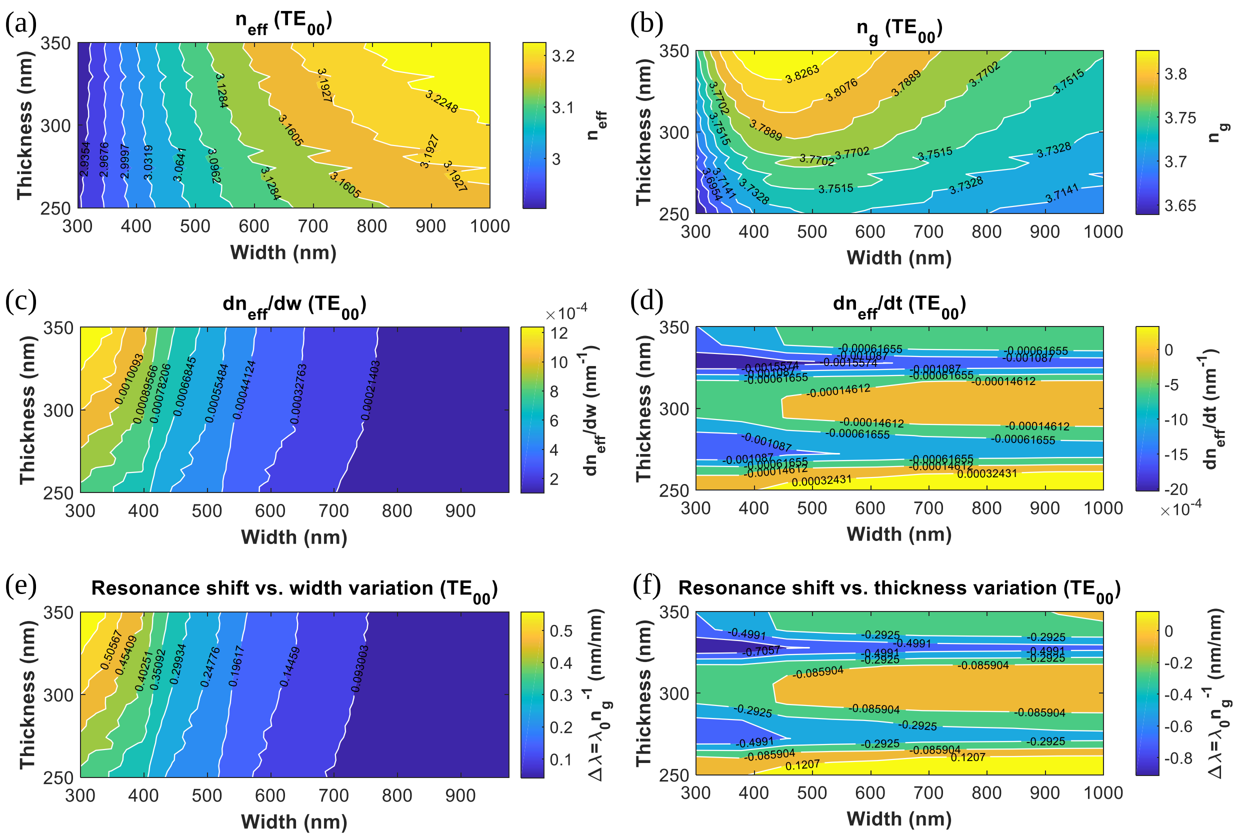

Supplementary Fig. 2 III-V/Si memristor optical mode calculations for (a) effective index (n_{eff}), (b) group index (n_g), (c) effective index change vs. waveguide width (dn_{eff}/dw), (d) effective index change vs. waveguide thickness (dn_{eff}/dt), (e) wavelength shift ($\Delta\lambda_0/dw$) vs. waveguide width, (f) wavelength shift vs. waveguide thickness ($\Delta\lambda_0/dt$).

$\lambda_0, n_{eff}, n_g, \Delta w,$ and Δt describe the free-space wavelength, effective index, group index, width variation, and thickness variation respectively. The resonant wavelength variation as a function of each dimension can be defined as: $\Delta\lambda_0 / \Delta w = (\lambda_0 / n_g)(dn_{eff} / dw)$ and $\Delta\lambda_0 / \Delta t = (\lambda_0 / n_g)(dn_{eff} / dt)$. Supplementary Fig. 2a – b shows the simulated effective and group index respectively. From this, we can determine the width sensitivity (dn_{eff}/dw) and thickness sensitivity (dn_{eff}/dt) as shown in Supplementary Fig. 2c – d. The single-mode III-V/Si memristor waveguides are designed to have dimensions of height = 300 nm, width = 500nm, etch depth = 170 nm, and GaAs thickness of 150 nm, thus resulting in effective index variations of $dn_{eff}/dw = 5.80 \times 10^{-4}$ /nm and $dn_{eff}/dt = -4.44 \times 10^{-5}$ /nm. The wavelength shift variation are $\Delta\lambda_0/dw = 0.2457$ nm/nm and $\Delta\lambda_0/dt = -0.0187$ nm/nm as shown in Supplementary Fig. 2e – f. If we assume a charge trap concentration of $Q_{TC} > 9\times 10^{19}$ cm^{-3}, which corresponds to a $\Delta n_{eff}^{III\text{-}V/Si} = 1.60 \times 10^{-3}$, the III-V/Si optical memristor can correct up to a $\Delta_{width} = 2.75$ nm or $\Delta_{height} = 36$ nm, which is quite significant. In an extreme case where a full $\Delta\varphi > 4\pi$ can be achieved with $\Delta n_g^{III\text{-}V/Si} = 13.7 \times 10^{-3}$ (supplementary note 5), we can significantly perform larger fabrication errors.

Supplementary Note 2: Transmission electron microscope (TEM) measurements of initial, set, and reset states.

We performed high resolution transmission electron microscope (HRTEM) imaging of initial, set, and reset states as shown in Supplementary Fig. 3a-c respectively. Visually, there does not appear to be any dielectric material degradation from the possibility of dielectric breakdown. The contrast differences between the 3 images may be due to variability in sample preparation and imaging. There seems to be no evidence of atomic diffusion or interfacial changes as indicated by the electron dispersive spectroscopy (EDS) line scans. This is furthermore verified by the 2D atomic composition mapping for each respective state as shown in Supplementary Fig. 4a-c. Supplementary Fig. 5a-c shows the quantitative 2D strain mapping of the oxide/Si interface for each respective state. It is observed that the set state has more stain locations compared to the initial and reset states. The process of resetting appears to remove some of the strain. Although strain can affect refractive index change, it remains to be determined how much this contributes to the large phase shifts we observe.

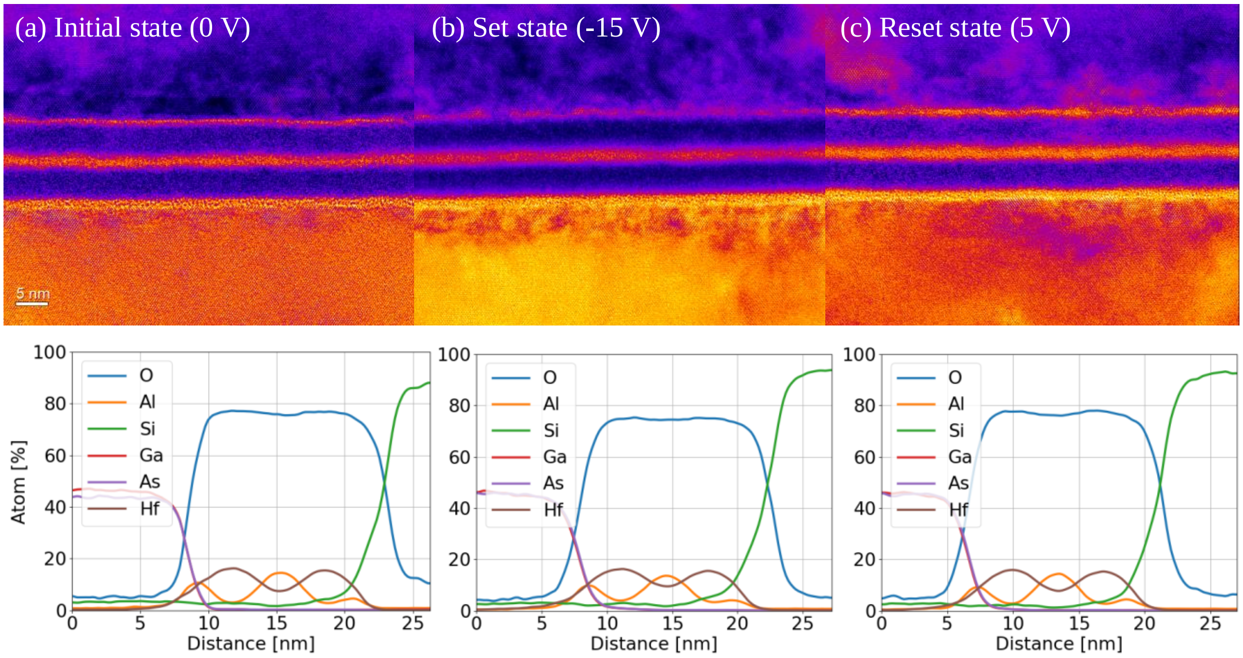

Supplementary Fig. 3 TEM imaging and EDS line scan of n-GaAs/Al_2O_3/HfO_2/Al_2O_3/HfO_2/ Al_2O_3/Si memristor structure in (a) initial state (0 V), (b) set state (-15 V), and (c) reset state (+ 5 V).

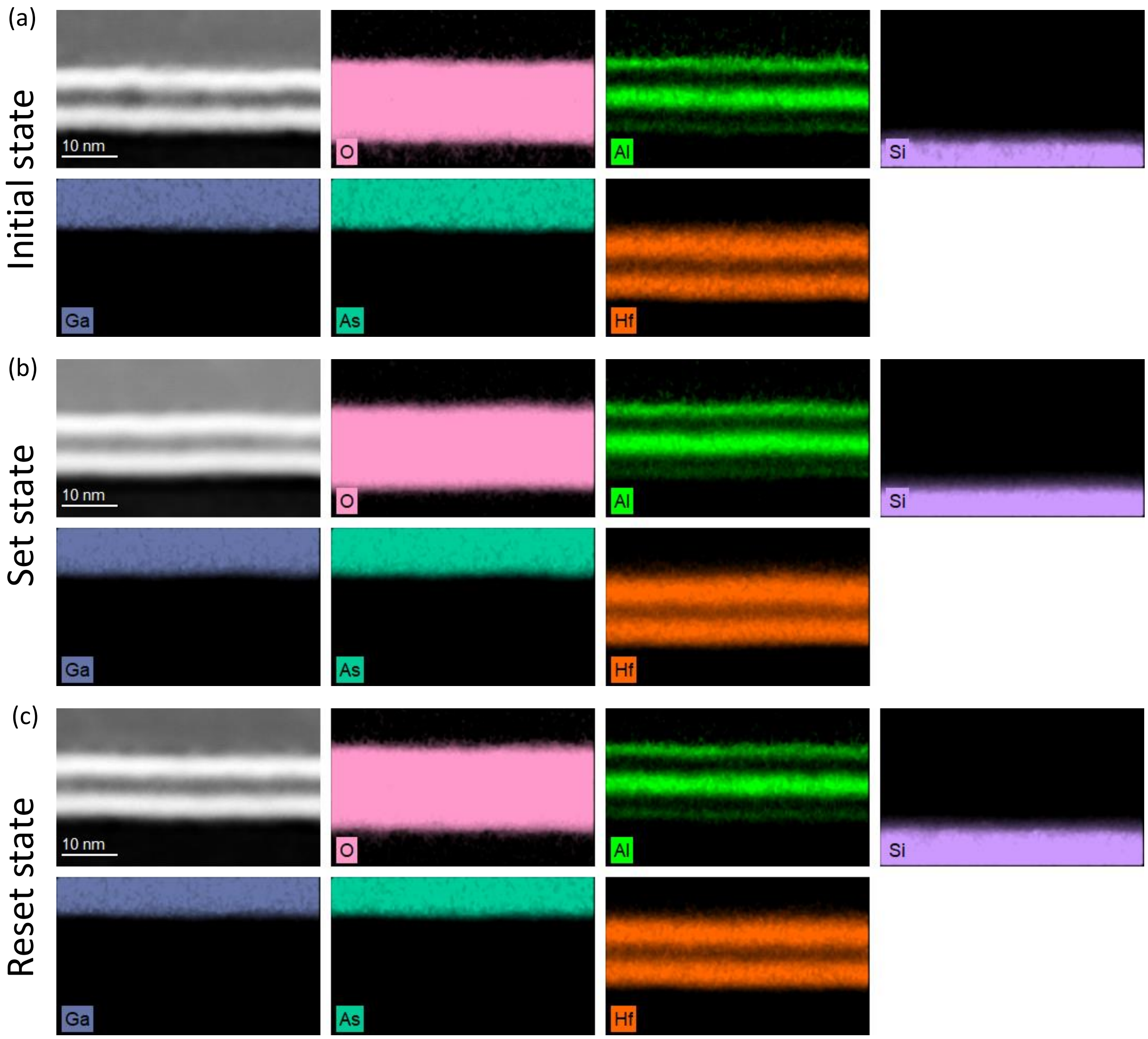

Supplementary Fig. 4. 2D atomic composition mapping for (a) initial state (0 V), (b) set state (-15 V), and (c) reset state (+ 5 V).

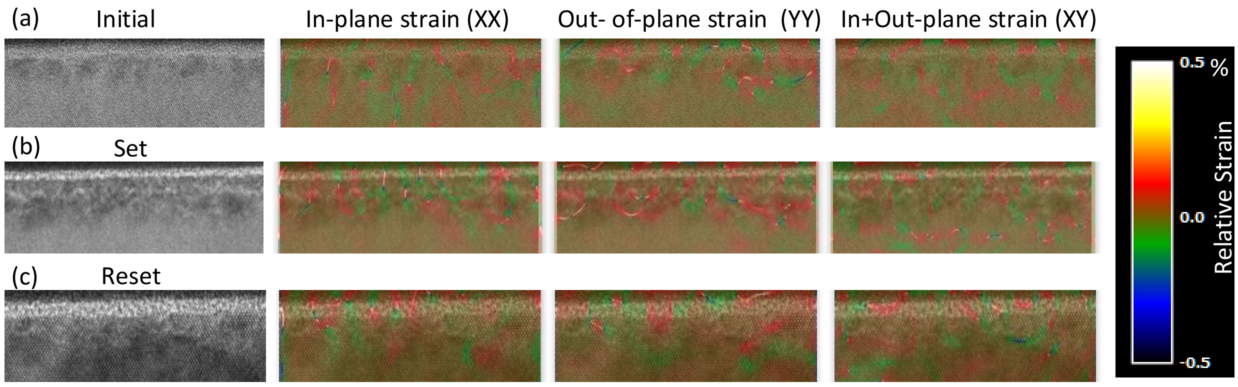

Supplementary Fig. 5. 2D strain mapping via geometrical phase analysis (GPA) for (a) initial state (0 V), (b) set state (-15 V), and (c) reset state (+ 5 V).

Supplementary Note 3: High speed S_{21} measurements for multiple non-volatile states.

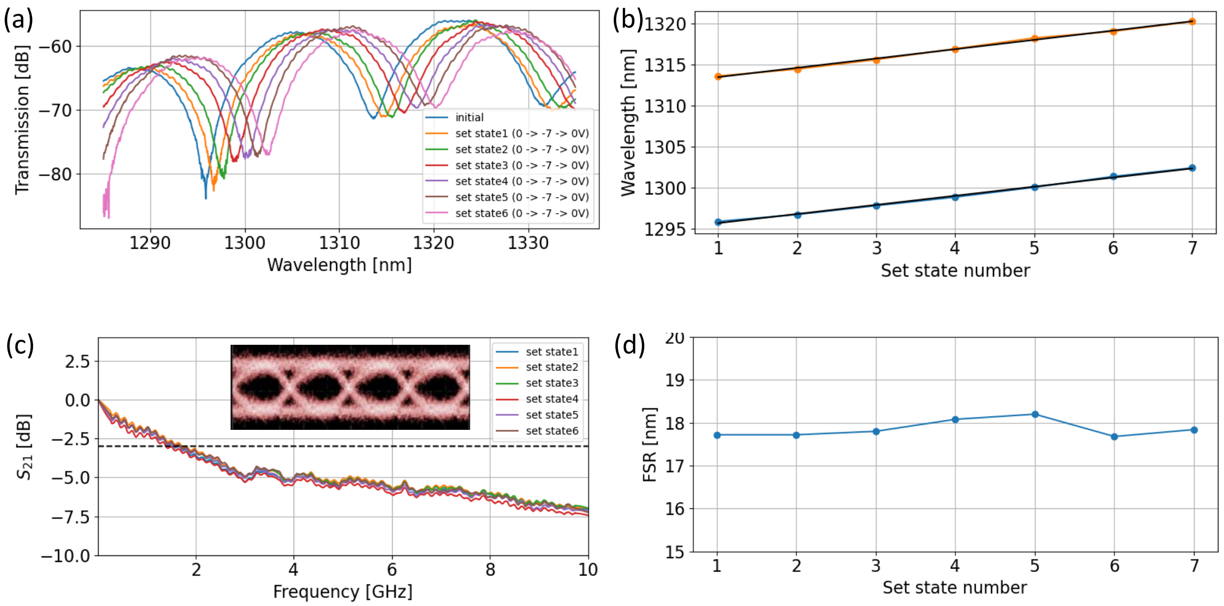

Supplementary Fig. 6. (a) Optical spectrum of 6 non-volatile set-states, (b) corresponding wavelength shifts, (c) small-signal S_{21} measurement for each set state (f_{3dB} ~ 1.9 GHz) and an eye-diagram at 4 Gbps, and (d) extracted FSR for each state.

Supplementary Note 4: An extreme case of phase shift tuning > 4π

Here is one particular device that exhibited a phase shift $\Delta\varphi > 4\pi$. This corresponds to a III-V/Si group index change of $\Delta n_g^{III\text{-}V/Si} = 13.7 \times 10^{-3}$ determined by the method mentioned in the main text. The same voltage cycling is performed (0 → - 21 → 0 → 15 →0 V) and an electrical and resistive hysteresis is observed in Supplementary Fig. 7a – b respectively. Applying a bias from 0 to -21 V results in a non-volatile wavelength shift of $\Delta\lambda_{non\text{-}volatile}$ ~ 40 nm wavelength shift with near negligible optical losses (Supplementary Fig. 7c). Ramping back down from -21 to 0 V does not shift the optical response back to the original state (Supplementary Fig. 7d) and has a non-volatile wavelength stability of ~ +/- 0.2 nm (35 GHz). This indicates a non-volatile phase shift of $\Delta\varphi > 4\pi$, at essentially 0 power consumption (recorded current = 10.4 pA at 0 V).

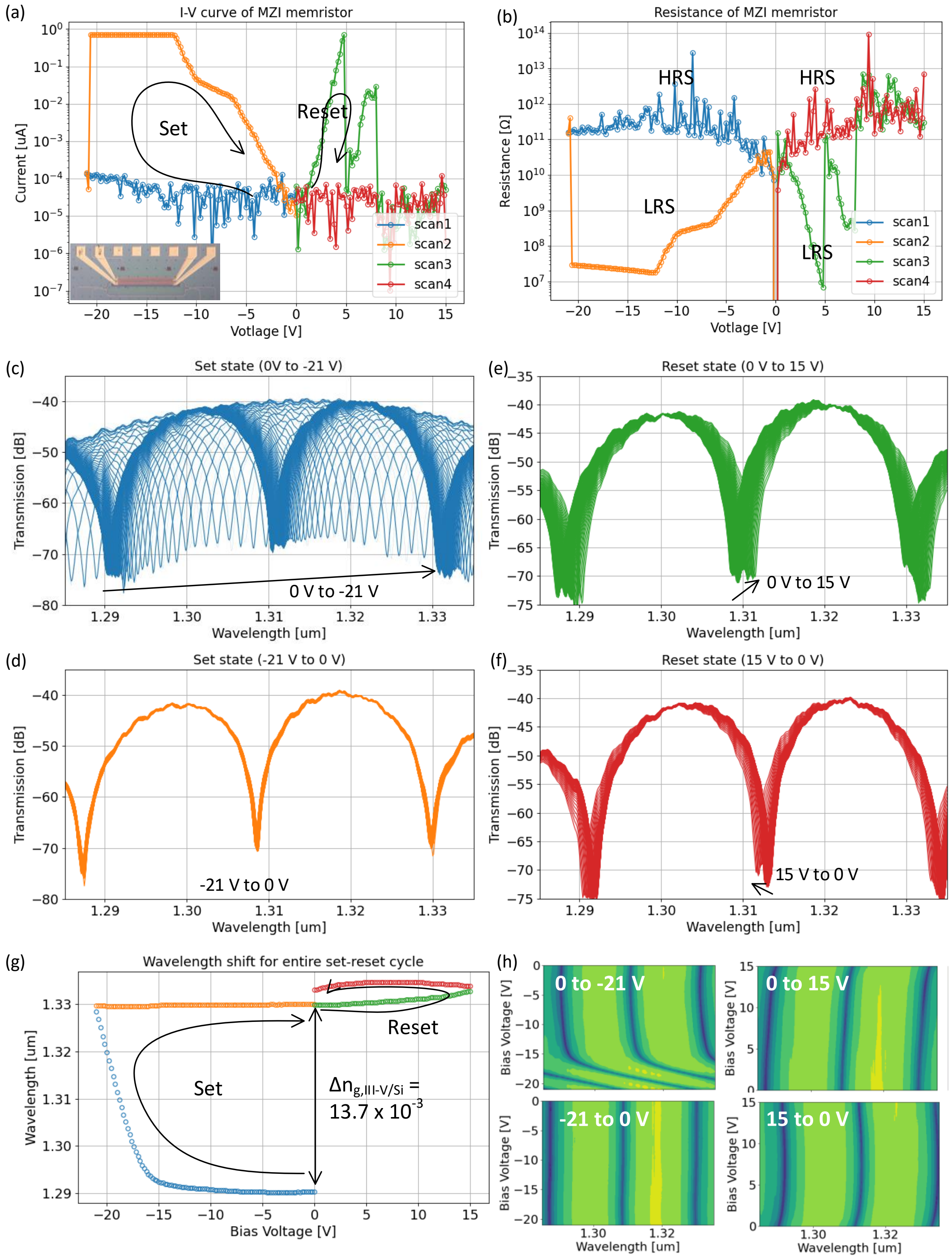

Supplementary Fig. 7. (a) Measured I-V hysteresis indicating non-volatile memristive set/reset states. (b) Corresponding resistance indicating regions of high-resistance-states (HRS) and low-resistance-states (LRS). Measured optical spectrum for (c) "set" (0 to -21 V), (d) turning off "set" (-21 to 0V), (e) "reset" (0

to 15 V), and (f) turning off "reset" (15 to 0 V). (g) Tracked resonance vs. voltage, (h) spectral evolution vs. voltage.

Supplementary Note 5: Insertion loss during non-volatile tuning

In order to determine the insertion loss of the MZI memristor during non-volatile tuning operation, we measured the optical spectrum of a reference, single-mode, straight waveguide (width=500 nm, height = 300 nm, etch depth = 170 nm, length = 870 µm). This is performed with a SLD and OSA. We have plotted the reference waveguide transmission and on top of MZI spectra under-going non-volatile tuning in the "set" regime. The insertion loss appears to be within 0.5 dB at 1310nm.

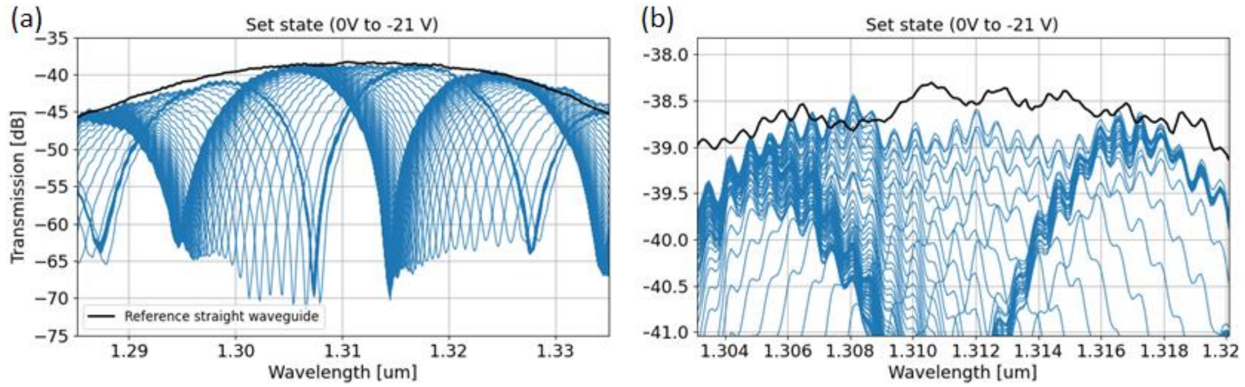

Supplementary Fig. 8. (a) Measured spectrum of MZI undergoing non-volatile tuning in the "set" regime with reference straight waveguide (black), (b) close-up of spectrum with π phase and reference waveguide indicating insertion loss remains < 0.5 dB.

Supplementary Note 6: Experimental setup for measuring non-volatile switching speeds

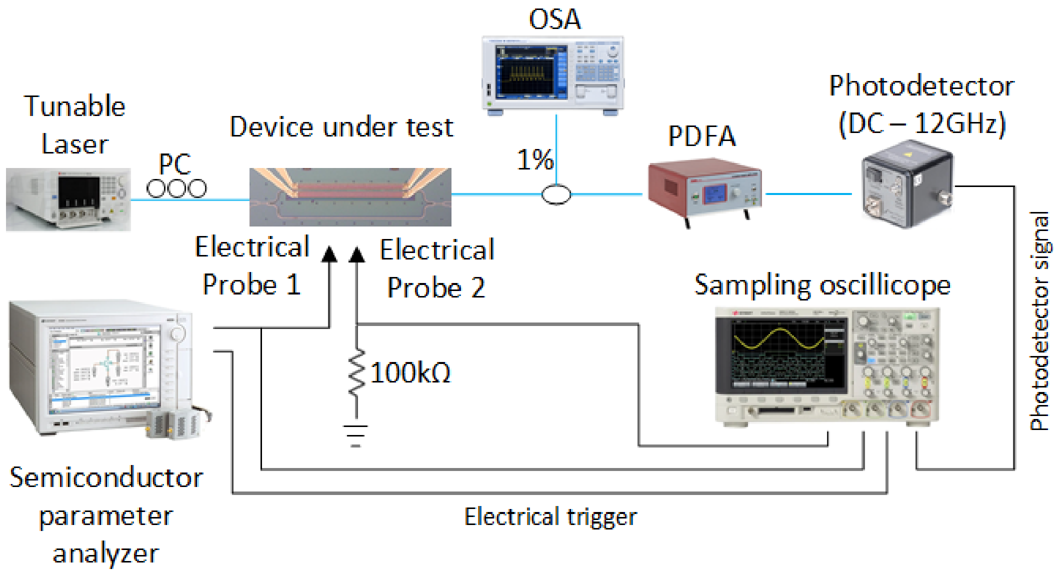

Supplementary Fig. 9. Diagram of measurement setup for measuring non-volatile switching speeds

Supplementary Note 7: Cyclability measurements

For one of the MZI devices, we were able to perform 3 cycles from set to reset before the device failed. The measured optical spectrum and corresponding I-V curves are shown below. In cycle 1, the blue represents the initial state and by performing a non-volatile set, ~ a π phase shift is achieved. Cycle 2 and Cycle 3 shows that we can repeatedly change the spectrum by a π phase shift multiple times.

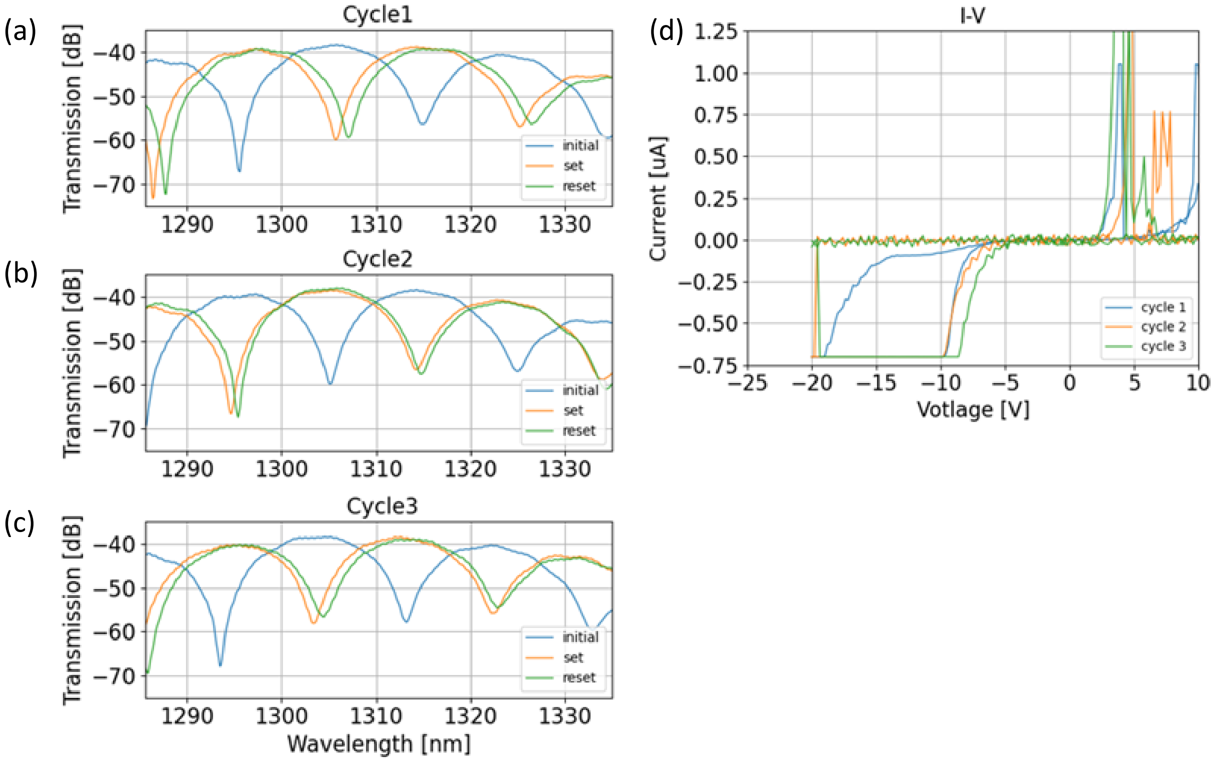

Supplementary Fig. 10. Cyclability of III-V/Si MZI memristor for 3 cycles (a) – (c) indicating a non-volatile π phase shift and (d) corresponding I-V hysteresis.

Supplementary Note 8: III-V/Si memristive photonic in-memory computing platform

Supplementary Fig. 11 (a) shows one possibility of an ONN architecture where III-V/Si memristors and silicon photonic computing elements are co-integrated together on a single heterogeneous substrate. Although this work solely focuses on MZIs and de-interleaver filters, they can extend to ring resonators and a variety of laser sources. This enables significant impacts to other ONN architectures such as ring resonator meshes[17] and hexagonal meshes[18]. The innovation described here comprises all the past demonstrated components needed for the proposed optical computing platform such as: Single-mode III-V/Si QD DFB lasers[19], SISCAP MZI[20,21], lossless light monitors[22,23], QD APDs[24,25], Ge APDs[26–29], programmable nonlinear activation functions[30,31], etc.) as well as the non-volatile III-V/Si memristor (this

work). Network weight training can be done via reliable, high-speed, power-efficient III-V/Si MZIs in a volatile push-pull operation (4 Gbps for our case [32] and > 30 Gbps with traveling wave electrode configuration [33]). For inferencing, we can employ the memristive state of the device and preferentially set the network weights and then remove the applied voltage for true "set-and-forget" 0 power consumption. The III-V/Si lossless light monitor [22,23] after each MZI allows for convenient and easy weight calibration without the use of complicated algorithms. The light monitors utilize internal trap mediated photo-carrier detection which induces no optical loss [22,23]. These detectors can be part of a feedback circuitry with the non-volatile III-V/Si memristor MZI for true in-memory optical computing. Supplementary Fig. 11 (b) shows a total tuning wavelength of 7.64 nm can be achieved from 0 V to -5 V and with 4 Gbps eye diagrams with Q = 4.6 [32]. For this particular MZI length, a phase shift of 0.85π can be achieved with a power consumption of 0.63 nW (~10,000,000 × improvement over thermal heaters [34]). In push-pull configuration, the wavelength tuning is higher with equivalent drive voltage. After network training is complete, one can adjust one arm of the III-V/Si MZI using the higher voltage, optical memristive effect for non-volatile inferencing.

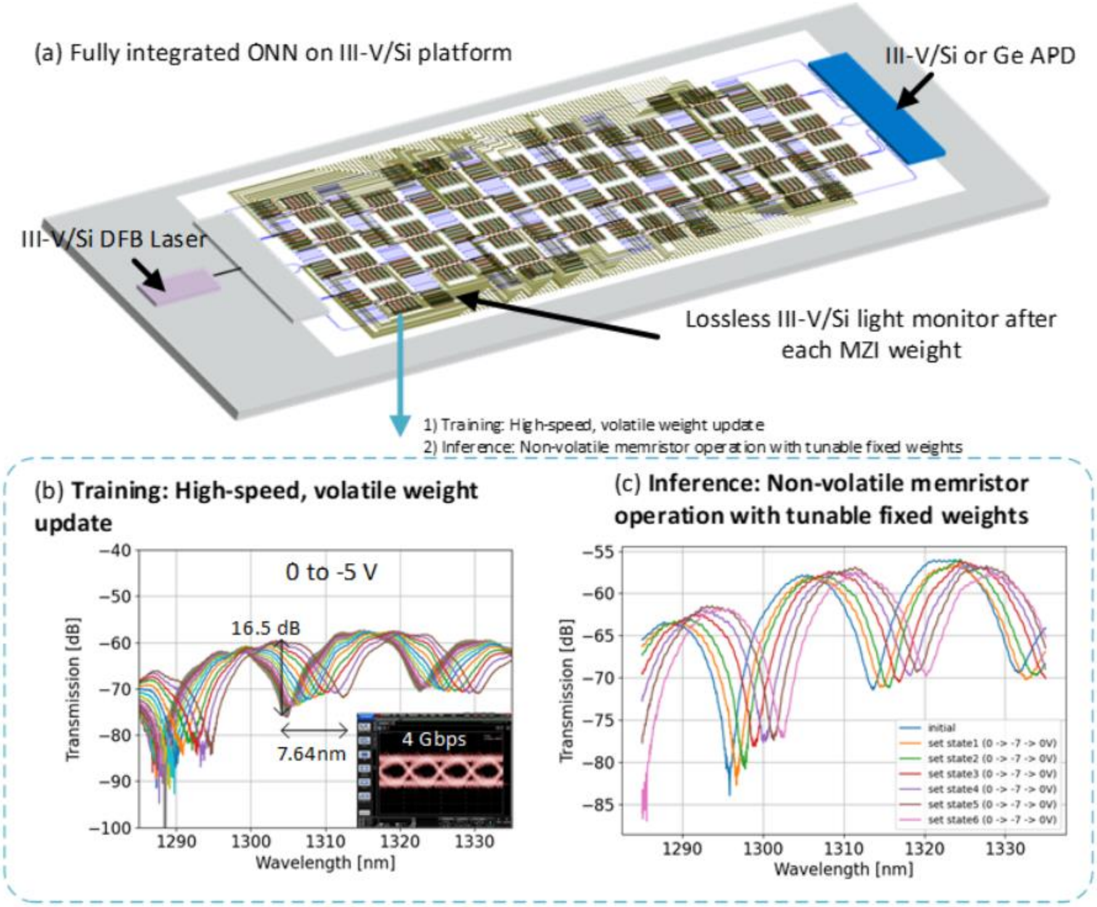

Supplementary Fig. 11. (a) Schematic of a fully-integrated ONN MVM mesh on a heterogeneous III-V/Si platform, (b) training via high-speed volatile weighting, and (c) use of non-volatile memristive MZI for inferencing operation after training procedures are finished.

Supplementary References

1. Atlas User Manual. (2022).
2. Bersch, E., Rangan, S., Bartynski, R. A., Garfunkel, E. & Vescovo, E. Band offsets of ultrathin high-κ oxide films with Si. *Phys. Rev. B* **78**, 085114 (2008).
3. Robertson, J. & Falabretti, B. Band offsets of high K gate oxides on III-V semiconductors. *J. Appl. Phys.* **100 (1)**, 014111 (2014).
4. Feng, Q., Yan, F., Luo, W. & Wang, K. Charge Trap Memory Based on Few-Layered Black Phosphorus. *Nanoscale* **8**, 2686-2692 (2016).
5. Zhang, Q. *et al.* Band offset and electron affinity of MBE-grown SnSe2. *Appl. Phys. Lett.* **112 (4)**, 042108 (2018).
6. Olivares, I., Parra, J. & Sanchis, P. Non-Volatile Photonic Memory Based on a SAHAS Configuration. *IEEE Photonics Journal* **13**, 9 (2021).
7. Spassov, D. *et al.* Charge Storage and Reliability Characteristics of Nonvolatile Memory Capacitors with HfO2/Al2O3-Based Charge Trapping Layers. *Materials* **15 (18)**, 6285 (2022).
8. Xiong, H. D. *et al.* Characterization of electrically active defects in high-k gate dielectrics by using low frequency noise and charge pumping measurements. *Microelectronic Engineering* **84 (9-10)**, 2230-2234 (2007).
9. She, M. Semiconductor Flash Memory Scaling. (University of California, Berkeley, 2003).
10. You, H.-C. *et al.* SONOS-Type Flash Memory Using an HfO2 as a Charge Trapping Layer Deposited by the Sol–Gel Spin-Coating Method. *IEEE Electron Device Letters* **27**, 653–655 (2006).
11. Zhang, Y. *et al.* Defect states and charge trapping characteristics of HfO2 films for high performance nonvolatile memory applications. *Appl. Phys. Lett.* **105**, 172902 (2014).
12. Cerbu, F. *et al.* Intrinsic electron traps in atomic-layer deposited HfO2 insulators. *Appl. Phys. Lett.* **108**, 22 (2016).
13. Palik, E. *Handbook of Optical Constants of Solids*. vols 1–5.
14. Chrostowski, L. & Hochberg, M. *Silicon Photonics Design: From Devices to Systems*. (Cambridge: Cambridge University Press, 2015).
15. Reed, T., Mashanovich, G., Gardes, Y. & Thomson, J. Silicon optical modulators. *nature photonics* **4**, 518-526 (2010).
16. Cheung, S. *et al.* Ultra-Power Efficient Heterogeneous III-V/Si De-Interleavers for DWDM Optical Links. in *IEEE 17th International Conference on Group IV Photonics (GFP)* 1–2 (2021). doi:10.1109/GFP51802.2021.9673963.
17. Ohno, S., Tang, R., Toprasertpong, K., Takagi, S. & Takenaka, M. Si Microring Resonator Crossbar Array for On-Chip Inference and Training of the Optical Neural Network. *ACS Photonics* **9**, 2614 (2022).
18. Bogaerts, W. *et al.* Programmable photonic circuits. *Nature* **586**, 207–216 (2020).
19. Liang, D. *et al.* High-performance quantum-dot distributed feedback laser on silicon for high-speed modulations. *Optica* **8**, 591–593 (2021).
20. Cheung, S. *et al.* Comparison of Al2O3 and HfO2 MOSCAP III-V/Si Power Splitters and (De-) Interleavers for DWDM Optical Links. in *Optical Fiber Communication Conference* (Optica Publishing Group, San Diego, CA, USA, 2022).
21. Cheung, S. *et al.* Ultra-power-efficient heterogeneous III–V/Si MOSCAP (de-)interleavers for DWDM optical links. *Photonics Research* **10**, A22–A34 (2022).
22. Srinivasan, S., Liang, D. & Beausoleil, R. In-situ light measurement in heterogeneous gain media. in *2021 27th International Semiconductor Laser Conference (ISLC)* 1–2 (2021). doi:10.1109/ISLC51662.2021.9615660.

23. Srinivasan, S., Liang, D. & Beausoleil, R. Non-invasive light monitoring for heterogeneous photonic integrated circuits. in *2021 IEEE Photonics Conference (IPC)* 1–2 (2021). doi:10.1109/IPC48725.2021.9593047.
24. Tossoun, B. *et al.* 32 Gbps heterogeneously integrated quantum dot waveguide avalanche photodiodes on silicon. *Opt. Lett.* **46**, 3821 (2021).
25. Tossoun, B. *et al.* Indium arsenide quantum dot waveguide photodiodes heterogeneously integrated on silicon. *Optica* **6**, 1277–1281 (2019).
26. Yuan, Y. *et al.* High Responsivity Si-Ge Waveguide Avalanche Photodiodes Enhanced by Loop Reflector. *IEEE Journal of Selected Topics in Quantum Electronics* **28**, 1–8 (2022).
27. Yuan, Y. *et al.* 64 Gbps PAM4 Si-Ge Waveguide Avalanche Photodiodes With Excellent Temperature Stability. *J. Lightwave Technol.* **38**, 4857–4866 (2020).
28. Yuan, Y. *et al.* OSNR Sensitivity Analysis for Si-Ge Avalanche Photodiodes. *IEEE Photonics Technology Letters* **34**, 321–324 (2022).
29. Peng, Y. *et al.* Demonstration of an Ultra-High-Responsivity All-Silicon Avalanche Photodetectors. in *2023 Optical Fiber Communications Conference and Exhibition (OFC)* 1–3 (2023). doi:10.1364/OFC.2023.W1A.2.
30. Yuan, Y. *et al.* Low-phase quantization error Mach–Zehnder interferometers for high-precision optical neural network training. *APL Photonics* **8**, 4 (2023).
31. Jha, A., Huang, C. & Prucnal, P. R. Reconfigurable all-optical nonlinear activation functions for neuromorphic photonics. *Opt. Lett.* **45**, 4819–4822 (2020).
32. Cheung, S. *et al.* Heterogeneous III-V/Si Non-Volatile Optical Memory: A Mach-Zehnder Memristor. in *2022 Conference on Lasers and Electro-Optics (CLEO)* 1–2 (San Jose, CA, 2022). doi:10.1364/CLEO_SI.2022.STu5G.6.
33. Dong, P., Chen, L. & Chen, Y. High-speed low-voltage single-drive push-pull silicon Mach-Zehnder modulators. *Opt. Express* **20**, 6163-6169 (2012).
34. Shen, Y. *et al.* Deep Learning with Coherent Nanophotonic Circuits. *Nature Photon* **11**, 441–446 (2017).
